# Longitudinal gut microbiome dynamics in rectal cancer: associations with neoadjuvant therapy and tumor regression

**DOI:** 10.1186/s12866-026-05859-y

**Published:** 2026-11-04

**Authors:** Jael Jentzsch, Stefan Wirtz, Maximilian Brunner, Silvia Ribback, Barbara Kneis, Klaus Weber, Axel Denz, Robert Grützmann, Robert Schierwagen, Olaf Tyc, Jonel Trebicka, Stephan Kersting, Melanie Langheinrich

**Affiliations:** 1 Department of Surgery, University Hospital Greifswald, 17475 Greifswald, Germany; 2 Department of Pediatrics, Klinikum Stuttgart, Olgahospital, 70174 Stuttgart, Germany; 3 Department of Internal Medicine I, University Hospital Erlangen, 91054 Erlangen, Germany; 4 Department of Surgery, University Hospital Erlangen, 91054 Erlangen, Germany; 5 Institute of Pathology, University Hospital Greifswald, 17475 Greifswald, Germany; 6 Department of Nephrology, University Hospital Erlangen, 91054 Erlangen, Germany; 7 Department of Internal Medicine B, University Hospital Münster, 48149 Münster, Germany; 8 Department of Internal Medicine II, Infectious Diseases, Goethe University Frankfurt, 60590 Frankfurt, Germany; 9 German Cancer Consortium (DKTK), partner site Frankfurt am Main, a partnership between DKFZ and University Hospital Frankfurt, Frankfurt, Germany

**Keywords:** Gut microbiome, Rectal cancer, Neoadjuvant therapy, Longitudinal analysis, 16S rRNA gene sequencing, Stool and tissue microbiome, Therapeutic response, Tumor regression, Complete response

## Abstract

**Background:**

The gut microbiome has emerged as a key modulator of colorectal cancer biology, influencing tumor progression and therapeutic response. However, most existing studies analyze colorectal cancer as a single entity despite biological and therapeutic differences between colon and rectal cancer. Consequently, longitudinal data linking microbiome dynamics to treatment response, particularly in rectal cancer, remain limited.

**Methods:**

In this prospective cohort study, 31 patients with rectal cancer were included, of whom 17 received neoadjuvant therapy and 14 underwent primary surgery. A total of 110 stool and tissue samples were collected at defined time points before, during, and after treatment. Microbial composition and diversity were analyzed using 16S rRNA gene sequencing. Alpha and beta diversity metrics, differential abundance analyses, and longitudinal comparisons were performed to assess treatment-related microbiome alterations.

**Results:**

Distinct microbial communities were observed between stool and tissue samples, with stool samples enriched in *Bacteroidetes* and tissue samples dominated by *Firmicutes*, *Actinobacteria*, and *Verrucomicrobia*. Neoadjuvant therapy was associated with a marked reduction in microbial diversity with no recovery before surgery, and community composition shifted significantly over the treatment course (patient-constrained PERMANOVA, *p* = 0.0016), with *Clostridium* XIVa becoming the dominant genus before surgery. Longitudinal analyses further revealed exploratory differences between patients with complete and incomplete tumor regression. Between diagnosis and surgery, patients achieving complete tumor regression (Dworak TRG 4) showed an increase in the propionate-producing genus *Phascolarctobacterium*, the only genus significant after correction for multiple testing, together with an exploratory rise in the butyrate-producing genus *Roseburia*. Higher *Bacteroides* in patients with incomplete regression was a descriptive observation that was not statistically significant (*p* = 0.35, q = 0.71). No genus-level differences between response groups were detected at baseline after correction for multiple testing; however, the small sample size limits the ability to exclude baseline differences.

**Conclusions:**

Rectal cancer treatment induces distinct and time-dependent alterations in the gut microbiome. Complete tumor regression was associated with an increase in short-chain fatty acid–producing taxa, led by the propionate producer *Phascolarctobacterium*, whereas higher *Bacteroides* in incomplete responders was a non-significant, exploratory observation. Given the growing role of organ-preserving strategies such as watch-and-wait, in which reliable identification of complete response is critical, these exploratory patterns warrant validation in larger cohorts.

**Supplementary information:**

The online version contains supplementary material available at https://doi.org/10.1186/s12866-026-05859-y.

## Background

The human gut microbiome, a complex network of trillions of microorganisms, plays a crucial role in maintaining health by regulating immune responses, metabolism, and inflammation. Its dysregulation has been linked to several diseases, including chronic diseases and cancer [1, 2]. Recent research has focused on understanding how the gut microbiome influences colorectal cancer (CRC) progression and patient responses to treatment [3–6]. Colorectal cancer (CRC), the third most common cancer worldwide, accounts for a substantial proportion of cancer-related deaths [7, 8]. Within CRC, rectal cancer represents approximately 30% of cases and poses unique challenges due to its anatomical location and the complexity of its treatment [9, 10]. Although patients with colorectal cancer are often considered as a single entity, rectal tumors differ substantially from colon cancer in both biology and treatment. In locally advanced rectal cancer, neoadjuvant chemoradiotherapy followed by surgery represents the standard of care. However, therapeutic response varies substantially: while some patients achieve only partial response, others develop a clinical or pathological complete response. For selected patients, a clinical complete response may enable a structured non-operative management or “watch-and-wait” strategy with organ preservation. This approach is particularly relevant for individuals who would otherwise require abdominoperineal resection (APR) with permanent stoma formation, which has a major impact on quality of life. Identifying biological factors associated with tumor response is therefore of considerable clinical relevance.

Emerging evidence suggests that the composition of the gut microbiome may affect the efficacy of cancer treatments such as chemotherapy and radiotherapy [11, 12]. Studies have demonstrated that specific microbial species can either enhance or impair the therapeutic response by modulating immune reactions and influencing inflammation [13, 14]. However, most research has focused on colorectal cancer in general, with relatively few studies specifically investigating rectal cancer. The relationship between the gut microbiome and treatment response in rectal cancer, especially in the context of neoadjuvant therapy, remains incompletely understood [15]. Recent studies have begun to explore this association, including analyses of both mucosal and fecal microbiota in relatively small, exploratory cohorts [16].

To address this gap, the present study investigates microbial alterations in patients undergoing rectal cancer treatment, with a particular focus on those receiving neoadjuvant therapy. By analyzing both stool and tissue samples across multiple clinically relevant timepoints, we aim to provide a comprehensive understanding of how therapy impacts the microbiome and to identify microbial signatures associated with better clinical outcomes. We hypothesize that longitudinal changes in the gut microbiome are associated with response to neoadjuvant therapy and tumor regression in rectal cancer.

## Methods

### Study design

This study population consists of 31 patients enrolled in the prospective Erlanger microbiome study, an observational trial approved by the local Ethics Committee. The participants were treatment-naive individuals scheduled for elective surgery or for neoadjuvant therapy followed by surgery, due to histologically confirmed rectal cancer. The decision to administer neoadjuvant therapy was made by the multidisciplinary tumor board in accordance with national guidelines, taking tumor stage and location into account. Patients with locally advanced low rectal cancer generally received neoadjuvant treatment, whereas those with early-stage tumors suitable for upfront resection underwent primary surgery. Of these, 17 patients received neoadjuvant therapy, while 14 underwent primary surgery. All patients underwent surgical resection, except for one patient who received neoadjuvant chemoradiotherapy but did not proceed to surgery and was therefore excluded from tissue-based analyses. The eligibility criteria excluded patients who had received antibiotic therapy within four weeks prior to treatment, had conditions significantly impacting gastrointestinal function, or required emergency surgery. All participants received identical mechanical oral bowel preparation, along with a standardized single dose of a third-generation cephalosporin and metronidazole approximately 30 min before surgery. Recruitment took place between 2018 and 2020. Tumor samples from the rectum, as well as non-tumor mucosal tissue samples from the proximal resection margin (colon) and distal resection margin (rectum), were collected intraoperatively. Furthermore, longitudinal stool samples were collected. In patients who underwent primary surgery, stool samples were taken at the time of initial tumor diagnosis (T0) and postoperatively (T3). In patients receiving neoadjuvant therapy, stool samples were collected at initial tumor diagnosis (T0), after completion of neoadjuvant therapy (T1), prior to surgery (approximately 8 weeks after completion of neoadjuvant therapy; T2), and postoperatively (T3). Postoperative stool samples obtained from patients with diverting ileostomy were collected via the stoma and coded as T3IS (ileostomy samples), allowing clear distinction. Tumor regression was assessed according to the Dworak grading system (TRG 0–4) based on histopathological evaluation of the resected specimen after neoadjuvant therapy. TRG 0 indicates no regression, TRG 1 minimal regression with predominance of tumor cells and fibrosis, TRG 2 moderate regression with predominant fibrosis and few tumor cells, TRG 3 near-complete regression with only scattered tumor cells in fibrotic tissue, and TRG 4 complete regression with no viable tumor cells. In this study, TRG 4 was defined as complete remission, whereas TRG 1–3 were categorized as incomplete remission. Throughout this study, complete response refers to pathological complete response (pCR), defined histopathologically as the absence of viable tumor cells in the resected specimen (TRG 4), and not to clinical complete response. Tumor regression grading therefore required a resection specimen and could be determined in 13 of the 17 neoadjuvant patients. In 4 neoadjuvant patients TRG could not be assessed, including one patient who declined surgery and from whom no resection specimen was available; these patients are reported as “unknown” in Table 1 and were excluded from regression-based tissue analyses. Because stool sampling was independent of surgery, longitudinal stool analyses could include patients for whom no resection specimen, and thus no TRG, was available; not every patient contributed a sample at every timepoint (for example, no postoperative [T3] stool sample was available for the patient who declined surgery). Tumor tissue for microbiome analysis was retained only when sufficient material remained after diagnostic histopathology, which took precedence over research sampling. Detailed clinical and lifestyle data, including smoking status, medication use, body mass index, dietary habits, use of dietary supplements, and symptoms at diagnosis, were collected using standardized questionnaires.

The study was conducted in accordance with the Declaration of Helsinki and approved by the Ethics Committee of the University of Erlangen (Protocol Number: 420_18 B). Informed consent was obtained from all participants.

### Sample processing and DNA purification

Stool samples were collected and stabilized prior to surgery and bowel preparation (preoperative stool) as well as postoperatively on days 5–7 (postoperative stool) using the Omnigene Gut system (DNA Genotek, Ottawa, Canada). Additionally, for patients undergoing neoadjuvant therapy, stool samples were also collected at the time of tumor diagnosis, after the completion of neoadjuvant therapy, and again before surgery (approximately 8–10 weeks after the end of the neoadjuvant therapy), following the same stabilization protocol. Samples were stored at -80°C until DNA extraction. DNA was extracted from stool using the PSP Stool DNA Kit according to the manufacturer’s instructions (Invitek Molecular, Berlin, Germany). Tissue samples from tumors and mucosal tissue were collected immediately after resection, suspended in RNA later buffer, and stored at -80°C. Sampling was performed on a separate back table, using a fresh sterile instrument set for each specimen, and macroscopic luminal content was avoided. All patients had undergone mechanical bowel preparation on the preceding day. The mucosal surface was not rinsed before sampling, in order to preserve loosely adherent mucosa-associated bacteria; the tissue samples therefore represent the mucosa-associated community, and a residual luminal contribution cannot be excluded. For every tissue-sampling procedure, an open RNAlater tube was carried through the collection as a field blank and passed on to the sequencing laboratory; no sequencing data are available for these field blanks, which therefore did not serve as a sequencing-based contamination control. DNA from tumor tissue and mucosal tissue at the proximal and distal resection margins was extracted using the Qiagen QIAamp Microbiome Kit (Qiagen, Hilden, Germany) following the manufacturer’s recommendations. Tissue samples were processed under a sterile laminar-flow bench with sterile instruments, and RNAlater was removed by washing with phosphate-buffered saline before extraction; during stool DNA extraction, all work surfaces were decontaminated with LookOut DNA Erase (Sigma-Aldrich) before each step. DNA was then quantified using a Qubit device (Thermo Fisher Scientific).

#### 16 S rRNA gene amplification and Illumina sequencing

The V3 + 4 region of the 16S rRNA gene was amplified using 10 ng of extracted DNA with degenerate region-specific primers (341F: 5’ – ACTCCTACGGGAGGCAGCAG – 3’; 806R: 5’ – GGACTACHVGGGTWTCTAAT – 3’), which included barcodes and Illumina flow cell adaptor sequences [17]. The amplification reaction consisted of 25 cycles for stool samples and 35 cycles for tissue samples (98°C for 15 s, 58°C for 20 s, 72°C for 40 s) using the NEBNext Ultra II Q5 Master Mix (New England Biolabs, Ipswich, MA). Amplicons were purified with Agencourt AMPure XP Beads (Beckman Coulter, Brea, CA), normalized, and pooled before sequencing on an Illumina MiSeq device with a 600-cycle paired-end kit, using standard Illumina HP10 and HP11 sequencing primers. Because stool and tissue were processed with different extraction kits and different amplification intensities, sample type is completely confounded with laboratory protocol; this limitation applies to every stool-versus-tissue comparison reported below.

#### Bioinformatic processing of the sequencing data

For bioinformatic analysis, sequencing reads were demultiplexed on the instrument using Cutadapt 3.6 [18]. All subsequent processing was performed in USEARCH 11.0.667 [19]. The terminal 15 bases of both forward and reverse reads were removed with fastx_truncate, and reads were merged and quality-filtered with the fastq_mergepairs and fastq_filter commands. Operational taxonomic units (OTUs) were generated with UPARSE [20] at a 97% identity threshold. Chimera detection and removal are integrated in the cluster_otus command and were not performed as a separate step. Taxonomy was assigned with SINTAX [21] at a confidence cutoff of 0.8 against the RDP 16 S rRNA training set v15. All reads were mapped to OTUs to generate an OTU table comprising 3,387 OTUs across 503 samples, of which 110 belonged to the present rectal cancer cohort.

Sequencing depth varied considerably across samples, with a median of 5,143 reads (interquartile range 2,118–14,552; maximum 45,651), and 68 of 110 samples contained fewer than 10,000 reads. Samples with fewer than 1,000 reads were excluded from all analyses; this removed 13 of 110 samples, leaving 97 for analysis. A complete sample-accounting overview, including samples per group and time-point/compartment, exclusions, and the exact samples and patients included in each analysis, is provided in Additional file 2: Table S1a–c. Good’s coverage supported this threshold: median coverage among included samples was 99.4%, whereas excluded samples ranged from 0% to 69% (Additional File 7). Good’s coverage did not differ across the longitudinal time-points (Kruskal–Wallis *p* = 0.86) despite a fourfold difference in median read count, suggesting comparable sampling completeness across time points. Singletons were removed, leaving 2,012 OTUs. Alpha diversity were calculated on the unfiltered, quality-controlled feature table; prevalence and variance filtering was not applied, because it removes rare taxa by design and invalidates richness estimation.

No-template controls were processed alongside the samples during library preparation and yielded no reads. Dedicated extraction blanks were not included. This is declared as a limitation. Normalization and transformation are specified separately for each analysis below.

### Microbiome analysis

Phylum- and genus-level composition and the core microbiome were assessed using MicrobiomeAnalyst [22], as means of the compartment-specific relative abundances across all stool samples. For these compositional summaries, features with fewer than 4 counts in at least 20% of samples were removed (1,889 features), followed by removal of the 10% of features with the lowest variance (interquartile range; 13 features), leaving 110 features. No taxa were removed on the basis of published contaminant lists. All diversity, differential-abundance and multivariate analyses were performed in R.

Alpha diversity was calculated after rarefaction to 1,300 reads (the minimum depth among the stool samples of the longitudinal analyses), using 256 subsampling iterations. The same depth was used for the compartment comparison; the four samples with fewer than 1,300 reads (two tissue and two ileostomy samples) were therefore not included in the rarefied analyses (93 of 97 samples). Because repeated samples from the same patient are not independent, alpha diversity was analysed using linear mixed-effects models of the form *diversity index ~ time point + (1 | patient)*. Pairwise contrasts between time points were estimated from estimated marginal means and corrected for multiple testing using the Benjamini-Hochberg approach. Beta diversity was assessed using Bray-Curtis dissimilarities calculated from log-transformed counts and visualised by principal coordinates analysis (PCoA). Differences in community composition between time points were tested by PERMANOVA, with 9999 permutations constrained within patients to account for repeated measurements. Homogeneity of multivariate dispersion was evaluated using betadisper. Jaccard and Aitchison distances were analysed as sensitivity analyses, and R² values are reported as measures of effect size. Differential abundance was assessed with MaAsLin2 using total-sum scaling and log transformation, with time-point modelled as a categorical fixed factor (diagnosis, T0, as the reference) and patient as a random effect. Response-dependent trajectories were assessed with a separate linear mixed-effects model in which genus-level relative abundances were log₁₀-transformed after adding a pseudocount of 10⁻⁴ and modelled as time-point × response, with patient as a random effect and time-point entered as a categorical factor with diagnosis (T0) as the reference, fitted by maximum likelihood with lme4; genera present in fewer than half the samples were excluded, leaving 57; interaction p-values were obtained from Wald z-tests and adjusted across genera by the Benjamini–Hochberg procedure. All taxon-level p-values are reported as Benjamini–Hochberg-adjusted q-values. Analyses were performed in R using vegan [23], phyloseq [24], lme4/lmerTest [25, 26], emmeans [27], MaAsLin2 [28], mixOmics [28, 29] and ggplot2 [30].

## Results

### Patient characteristics

A total of 31 patients with rectal cancer were included in this study, comprising 17 who received neoadjuvant therapy and 14 who underwent primary surgery without prior treatment. The age of the study participants ranged from 44 to 87 years. The median age did not differ significantly between groups, with the majority of patients aged between 60 and 79 years. Both cohorts showed a similar sex distribution. Body mass index (BMI), alcohol consumption, smoking status, and meat intake were comparable across groups, with no statistically significant differences (all *p* > 0.05). Most patients reported symptoms at diagnosis, with hematochezia being the most frequent.

Within the neoadjuvant group 15/17 patients received a neoadjuvant long-course chemoradiation with 5-FU and oxaliplatin, while two patients were treated with neoadjuvant chemotherapy alone. Surgical approach differed slightly between groups: minimally invasive surgery was more common in the non-neoadjuvant group, whereas open surgery predominated among patients after neoadjuvant therapy. Pathological tumor and nodal stages were evenly distributed. Tumor regression was graded according to Dworak with reference to the invasive tumor component. Regression grading was evaluable in 13 of the 17 neoadjuvant patients. Complete regression (TRG 4) was achieved in four patients ( 4 of 17) neoadjuvant patients (23.5%), corresponding to 4 of 13 patients with evaluable regression grading (30.8%). Three of these patients were staged ypT0N0. In the fourth patient, two distinct findings coexisted: the invasive carcinoma showed complete regression, with a carcinoma-free resection specimen, and, separately, a 3 mm focus of intramucosal high-grade neoplasia (adenocarcinoma in situ) was present, resulting in a stage of ypTisN0. As pathological complete response is conventionally defined as ypT0N0, this case represents complete regression of the invasive tumor but not a pCR in the strict sense; response analyses therefore refer to Dworak grading rather than pCR. All resected cases achieved an R0 status, and no significant differences in M-stage were observed between groups. Overall, the two cohorts were clinically comparable with respect to major demographic and clinical characteristics (Table 1).

**Table 1:** Clinicopathological characteristics

Patient characteristics	neoadjuvant therapy yes *n* (%)	neoadjuvant therapy no *n* (%)	*p*-value
Age o < 60 o ≥ 60–79 o ≥ 80	5 (29.4) 11 (64.7) 1 (5.9)	7 (50) 5 (35.7) 2 (14.3)	0.2656
Sex o Female o Male	5 (29.4) 12 (70.6)	3 (21.4) 11 (78.6)	0.9258
BMI o $\:\mathrm{U}\mathrm{n}\mathrm{d}\mathrm{e}\mathrm{r}\mathrm{w}\mathrm{e}\mathrm{i}\mathrm{g}\mathrm{h}\mathrm{t}\:<$ 18.5 o $\:\mathrm{N}\mathrm{o}\mathrm{r}\mathrm{m}\mathrm{a}\mathrm{l}\:\mathrm{w}\mathrm{e}\mathrm{i}\mathrm{g}\mathrm{h}\mathrm{t}\ge\:$ 18.5–24.99 o Overweight$\:\:\ge\:$ 25–29.99 o Obesity class I$\:\:\ge\:$ 30–34.99 o $\:Obesity\:class\:II\:\ge\:$ 35–39.99 o Obesity class III$\:\:\ge\:$ 40	1 (5.9) 5 (29.4) 8 (47.1) 2 (11.8) 1 (5.9) 0	0 5 (35.7) 5 (35.7) 2 (14.3) 1 (7.1) 1 (7.1)	0.9947
Alcohol o none o * <* 1/ week o 1–2/ week o 3–4/ week o 5–6/ week o daily o unknown	2 (11.8) 4 (23.5) 3 (17.6) 2 (11.8) 0 5 (29.4) 1 (5.9)	1 (7.1) 7 (50) 0 0 2 (14.3) 3 (21.4) 1 (7.1)	0.7819
Meat consumption o none o 1–2/ week o 3–4/ week o 5–6/ week o daily o unknown	0 2 (11.8) 3 (17.6) 2 (11.8) 9 (52.9) 1 (5.9)	0 2 (14.3) 2 (14.3) 3 (21.4) 4 (28.6) 3 (21.4)	0.6883
Smoking o yes o no o unknown	4 (23.5) 12 (70.6) 1 (5.9)	3 (21.4) 11 (78.6) 0	0.9697
Medication o none o 1–3 o 3–5 o > 5	8 (47.1) 8 (47.1) 1 (5.9) 0	7 (50) 4 (28.6) 1 (7.1) 2 (14.3)	0.7403
Symptoms o yes o no o unknown	13 (76.5) 3 (17.6) 1 (5.9)	10 (71.4) 4 (28.6) 0	0.8143
Hematochezia o yes o no o unknown	10 (58.8) 6 (35.3) 1 (5.9)	7 (50) 7 (50) 0	0.7854
Type of operation o open o minimally invasive o no operation	9 (52.9) 7 (41.2) 1 (5.9)	4 (28.6) 10 (71.4) 0	0.3109
T-stage o Tis o 0 o 1 o 2 o 3 o unknown	1 (5.9) 4 (23.5) 1 (5.9) 2 (11.8) 8 (47.1) 1 (5.9)	0 0 6 (42.9) 2 (14.3) 6 (42.9) 0	0.3410
N-stage o 0 o 1 o 2 o unknown	11 (64.7) 4 (23.5) 1 (5.9) 1 (5.9)	12 (85.7) 1 (7.1) 1 (7.1) 0	0.6313
Tumor regression o 1 o 2 o 3 o 4 o unknown	1 (5.9) 2 (11.8) 6 (35.3) 4 (23.5) 4 (23.5)	- - - - -	-
R-status o locally R0	16/16 (100%)	14/14 (100%)	1.000
M-status o 0 o 1	15 (88.2) 2 (11.8)	13 (92.9) 1 (7.1)	1.000

### Microbiome composition and diversity in stool and tissue samples

**Fig. 1:**
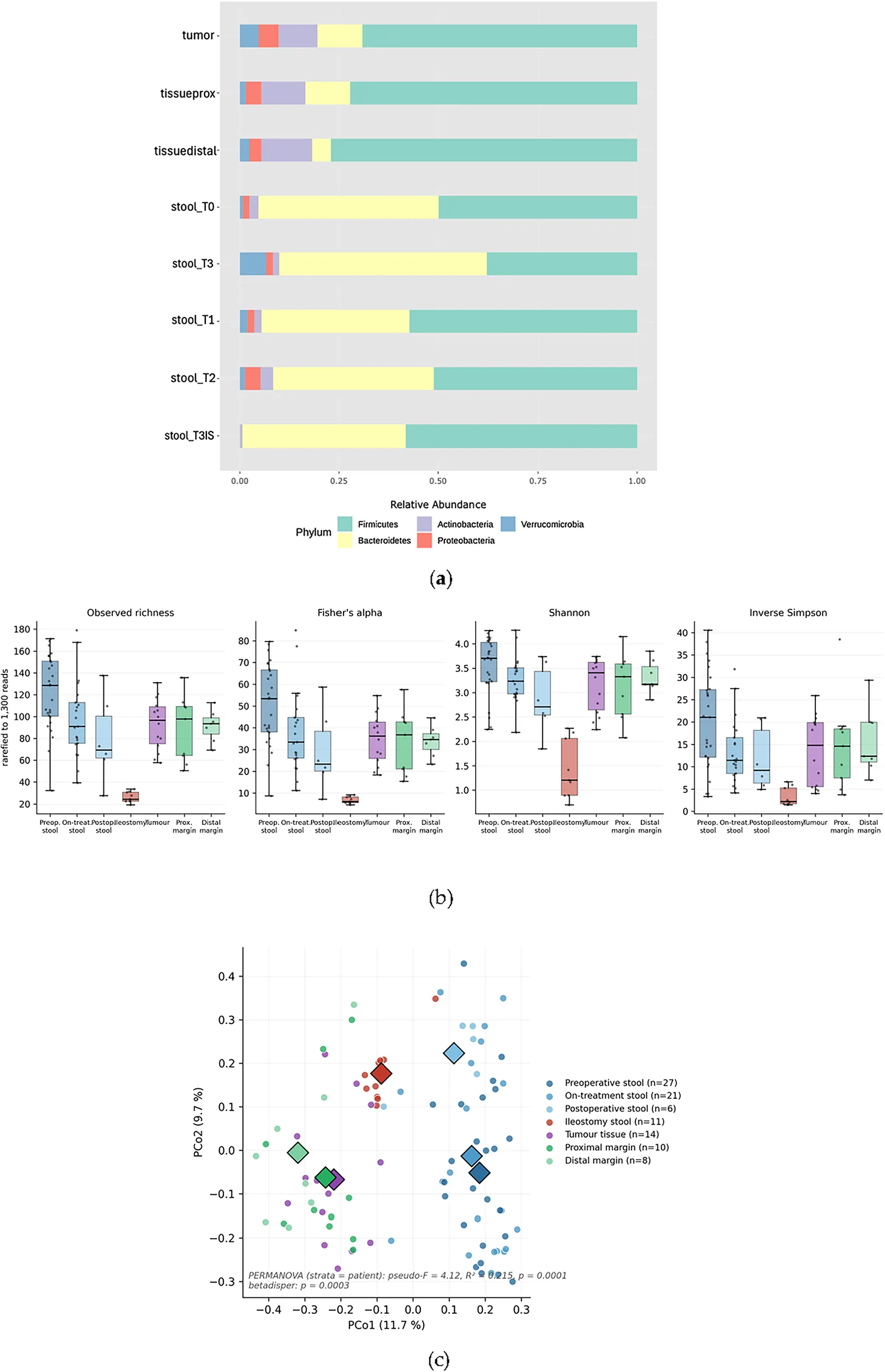
Microbiome composition and diversity across compartments (31 patients after quality control; panel (**a**) phylum level, 97 samples; panels (**b**) and (**c**) OTU level, rarefied to 1,300 reads, 93 samples). **a** Phylum-level composition by compartment and time-point. **b** Alpha diversity (observed richness, Fisher’s alpha, Shannon, inverse Simpson) across seven compartments; linear mixed-effects models with patient as random effect, all *p* ≤ 1.4 × 10⁻⁴; ileostomy-derived stool showed the lowest diversity. **c** Principal coordinates analysis of Bray–Curtis dissimilarities with group centroids; PERMANOVA with patient-constrained permutations (pseudo-F = 4.12, R² = 0.215, *p* = 0.0001, 9,999 permutations); dispersion differed between compartments (*p* = 0.0003)

First we conducted abundance profiling to characterize the microbiome across all 31 patients. The dominant phyla detected were *Firmicutes*, *Bacteroidetes*, *Actinobacteria*, *Verrucomicrobia*, and *Proteobacteria* (Fig. 1a). Differences in composition were observed between stool and tissue samples. Across all stool samples, *Firmicutes* (50%) and *Bacteroidetes* (44%) were most prevalent, whereas tissue samples were dominated by *Firmicutes* (73%) (Additional File 1: Fig. S1 and S6), with substantial variation between individual samples. To further explore taxonomic differences, we examined microbial composition at the genus level. Tumor tissue was dominated by *Gemmiger* (12%), *Blautia* (11%), *Bacteroides* (8%), and *Anaerostipes* (7%), whereas the proximal resection-margin tissue showed a higher relative abundance of *Lachnospiracea incertae sedis* (25%), followed by *Blautia* (12%), *Gemmiger* (8%), and *Bacteroides* (8%). Distal tissue of the resection margin was characterized by *Gemmiger* (19%), *Lachnospiracea incertae sedis* (17%), *Blautia* (15%), and *Anaerostipes* (8%) (Additional file 2: Fig. S2).

Alpha diversity across compartments. All four indices differed significantly between compartments (observed richness *p* = 2.7 × 10⁻¹³, Fisher’s alpha *p* = 1.3 × 10⁻¹¹, Shannon *p* = 3.0 × 10⁻¹⁴, inverse Simpson *p* = 1.4 × 10⁻⁴; likelihood-ratio tests, patient as random effect; 93 samples from 31 patients, rarefied to 1,300 reads). Compared with preoperative stool (median observed richness 128.6, Shannon 3.7), diversity was lower in every other compartment and by far the lowest in ileostomy-derived stool (24.5, 1.2; both q < 0.0001), whereas postoperative stool with colonic passage was intermediate (69.5, 2.7) (Fig. 1b). Pooling postoperative and ileostomy samples would therefore overstate the postoperative decline.

Beta diversity across compartments. PERMANOVA with patient-constrained permutations showed significant separation (pseudo-F = 4.12, R² = 0.215, *p* = 0.0001, 9,999 permutations). Multivariate dispersion also differed between compartments (*p* = 0.0003), so the separation cannot be attributed to differences in group centroids alone (Fig. 1c).

### Comparative microbiome shifts in neoadjuvant and non-neoadjuvant-treated patients

Microbiome composition differed between non-neoadjuvant (primary surgery) and neoadjuvant-treated patients already at baseline. We analyzed the fecal samples of all 31 patients. The preoperative microbiome analysis of the non-neoadjuvant cohort revealed the following descending order of phyla: *Bacteroidetes* (50%), *Firmicutes* (43%), *Proteobacteria* (3%), *Actinobacteria* (2%), *Fusobacteria* (2%). In the neoadjuvant-treated cohort: *Firmicutes* were the most dominant taxa, with an abundance of 61%, followed by *Bacteroidetes* (34%), *Proteobacteria* (2%), *Actinobacteria* (2%), *Verrucomicrobia* (1%).

Next, we analyzed the core microbiome at the genus level. Genera with a sample prevalence above 50% and a relative abundance above 0.01% were identified. The core analysis revealed the genera *Bacteroides* and *Clostridium XIVa* in both cohorts as core taxa across all samples before and after surgery. Postoperatively, *Akkermansia* was prevalent in both groups, while *Enterococcus* belongs to the core taxa set in the non-neoadjuvant cohort.

At baseline, before any treatment, none of the four alpha-diversity indices differed between neoadjuvant and non-neoadjuvant patients (exact Mann–Whitney on rarefied data; smallest unadjusted *p* = 0.081, all Benjamini–Hochberg q = 0.11; 14 versus 13 patients). At the phylum level, however, the dominant taxa differed, with higher Firmicutes and lower Bacteroidetes in neoadjuvant patients (medians 60% vs. 37% and 34% vs. 44%; exact Mann–Whitney *p* = 0.019 and 0.049), consistent with the two groups representing different clinical populations. The overall genus-level community did not separate significantly (PERMANOVA on log-transformed Bray–Curtis dissimilarities, pseudo-F = 0.88, R² = 0.034, *p* = 0.57). Treatment-effect analyses were therefore performed longitudinally within patients, where each patient serves as their own baseline.

#### Impact of neoadjuvant therapy on microbial diversity

During neoadjuvant therapy, microbial richness decreased in fecal samples significantly. All neoadjuvant treated rectal cancer patients were included in this analysis. Pre-treatment samples were dominated by *Bacteroides* (18%), *Prevotella* (13%), *Oscillibacter* (9%), *Faecalibacterium* (5%), and *Phascolarctobacterium* (5%). Post-treatment samples showed an increase in *Faecalibacterium* (10%), *Roseburia* (7%), and *Clostridium XIVa* (6%), while *Oscillibacter* (5%) decreased.

At the phylum level, the relative abundance of *Firmicutes* decreased progressively over time (57%, 55%, 52%), with the lowest levels observed in postoperative (T3) samples. In contrast, the relative abundance of *Bacteroidetes* increased gradually during the course of therapy and reached its highest proportion postoperatively (60%; T3). At genus level, *Bacteroides* accounted for 18% of reads at diagnosis, around 20% after neoadjuvant therapy and 16% before surgery, showing no consistent change during treatment; response-dependent analyses, including *Bacteroides*, are described in Section "Longitudinal stool microbiome and tumor regression" Postoperative (T3) samples are reported here descriptively and are not part of the neoadjuvant-therapy comparison; in these samples, *Bacteroides* increased to 52%.

**Fig. 2:**
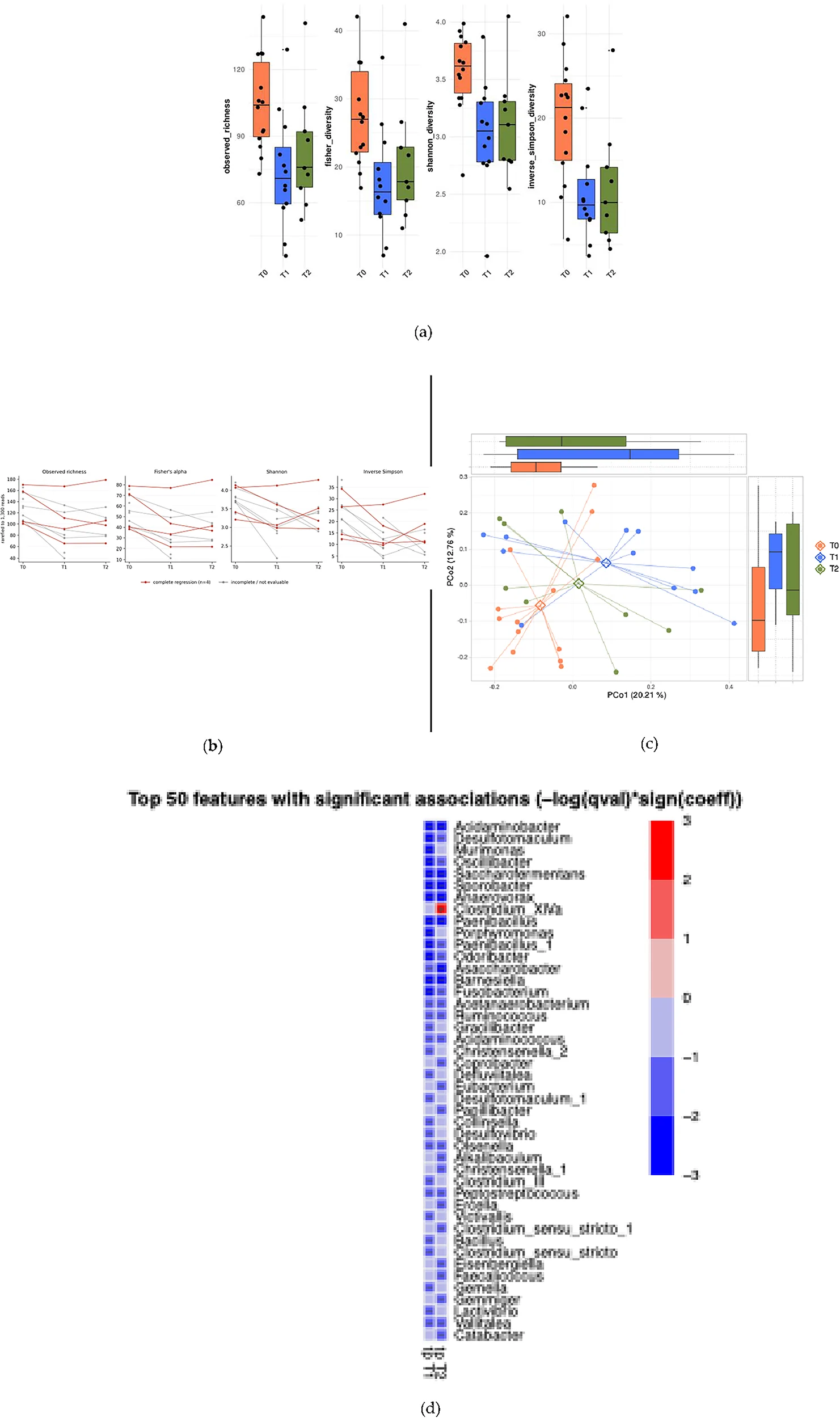
Longitudinal changes in the fecal microbiome during neoadjuvant therapy (35 samples from 17 patients; T0 *n* = 14, T1 *n* = 12, T2 *n* = 9). **a** Alpha diversity at each time-point for four indices (observed richness, Fisher’s alpha, Shannon, inverse Simpson). All indices differed across time-points in a linear mixed-effects model with patient as a random effect (Benjamini–Hochberg-adjusted *p* = 0.0009 to 0.0026); the decline occurred between T0 and T1, with no difference between T1 and T2. **b** Individual patient trajectories for the same indices (rarefied minimun sequencing depth); patients with complete regression in red. **c** Principal coordinates analysis of log-transformed Bray–Curtis dissimilarities (PERMANOVA with patient-constrained permutations, *p* = 0.0016, R² = 0.067; dispersion *p* = 0.11). **d** MaAsLin2 heatmap of the 50 genera most strongly associated with time-point (patient as random effect); Clostridium XIVa was the only genus with a positive association (q = 0.051)

Alpha diversity declined significantly during neoadjuvant therapy. In a linear mixed-effects model with patient as a random effect, all four indices differed significantly across time-points (observed richness *p* = 0.0009, Fisher’s alpha *p* = 0.0009, Shannon *p* = 0.0026, inverse Simpson *p* = 0.0026; Benjamini–Hochberg adjusted; 35 samples from 17 patients) (Fig. 2a). Time-point–specific contrasts localize the change to the interval between diagnosis and the end of neoadjuvant therapy. Diversity was lower at T1 than at T0 for every index (observed richness q = 0.0007, Fisher’s alpha q = 0.0007, Shannon q = 0.0043, inverse Simpson q = 0.0053) and remained lower at T2 (q = 0.0019, 0.0018, 0.0081 and 0.0064, respectively). No index differed between T1 and T2 (all q ≥ 0.72), indicating that diversity did not recover before surgery (Fig. 2b). Good’s coverage did not differ across time-points (*p* = 0.86). In beta diversity, community composition differed across time-points (PERMANOVA on log-transformed Bray–Curtis dissimilarities with patient-constrained permutations, *p* = 0.0016, R² = 0.067; dispersion test *p* = 0.11) (Fig. 2c). The rarefied analysis gave a consistent result (*p* = 0.0094) but with significant dispersion differences and is reported as a sensitivity analysis only (Additional file 6: Fig. S3).

Differential abundance was assessed with MaAsLin2, using patient as a random effect and time-point as a fixed factor. Seven genera were significantly less abundant after neoadjuvant therapy than at diagnosis: *Acidaminobacter* (q = 0.039 at both T1 and T2), *Desulfotomaculum*,* Murimonas*,* Oscillibacter*,* Saccharofermentans* and *Sporobacter* (all q = 0.039 at T1) and *Anaerovorax* (q = 0.045 at T1). *Clostridium XIVa* was the only genus showing a positive association with time, narrowly missing the significance threshold (q = 0.051 at T2) (Fig. 2d). In a patient-level multilevel sPLS-DA on centered log-ratio–transformed genus abundances (30 samples with complete pairing), Clostridium XIVa was the dominant contributor to the second component, which separated pre-surgical (T2) samples from earlier time-points, independently recovering the genus identified by MaAsLin2.

The core microbiome of stool at diagnosis was dominated by *Bacteroides*, *Oscillibacter*, *Faecalibacterium*, and *Clostridium XIVa*, with these genera showing the highest prevalence across detection thresholds (Additional file 1: Fig. S5a). These genera were highly prevalent in the gut microbiome before treatment, reflecting a more diverse microbial community. After neoadjuvant therapy, the prevalence of *Oscillibacter* and *Lachnospiracea incertae sedis* declined markedly, while *Faecalibacterium* and *Clostridium XIVa* remained highly prevalent (Additional file 1: Fig. S5b). *Bacteroides* remained highly prevalent. By the time immediately prior to surgery (T2), *Clostridium XIVa* had become the most prevalent genus, reflecting a marked shift in the gut microbiome (Additional file 1: Fig. S5c). *Prevotella* emerged at this stage, while *Oscillibacter* and *Lachnospiracea incertae sedis* were less prevalent at higher detection thresholdes.

#### Longitudinal stool microbiome and tumor regression

Regression grading was evaluable in 13 of the 17 neoadjuvant patients. Four achieved complete regression (Dworak TRG 4) and nine did not. At diagnosis, no genus differed between the two response groups after correction (smallest q = 0.96), *Roseburia* included (q = 0.99); no baseline genus-level difference between later responders and non-responders was detected, although with four complete responders the analysis has limited power to exclude such a difference.

**Fig. 3:**
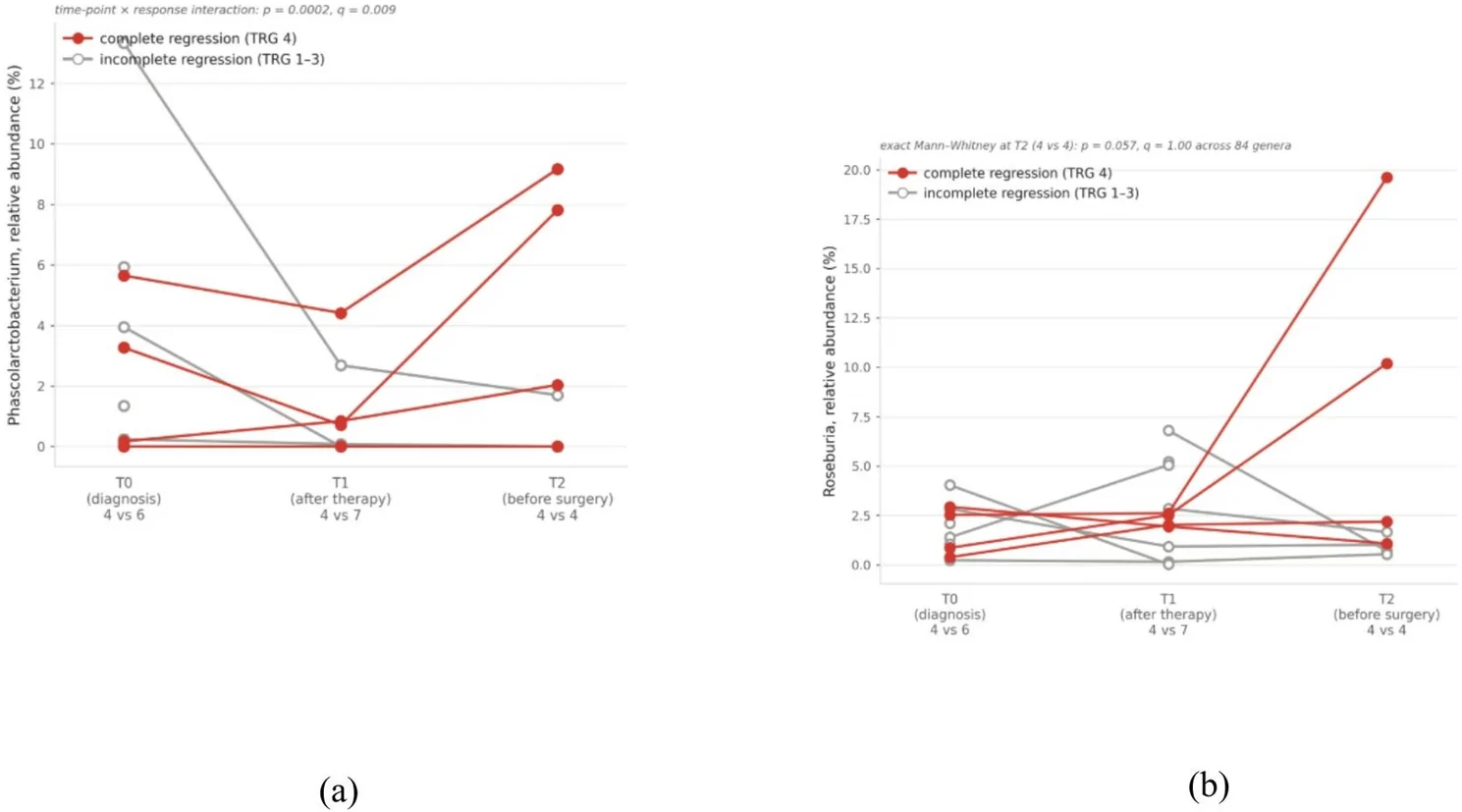
Individual trajectories of *Phascolarctobacterium* (**a**) and *Roseburia* (**b**) in the 13 neoadjuvant patients with evaluable tumor regression (complete *n* = 4, incomplete *n* = 9; genus level). Numbers below each time point give the patients contributing a sample. **a** Phascolarctobacterium: time-point × response interaction, linear mixed-effects model with patient as random effect, coefficient − 1.70, *p* = 0.0002, Benjamini–Hochberg-adjusted q = 0.009 across 57 genera. **b** Roseburia: time-point × response interaction *p* = 0.085, q = 0.33 (57 genera); in addition, exact Mann–Whitney test at T2 (4 vs. 4 patients), *p* = 0.057, q = 1.00 (84 genera)

*Phascolarctobacterium* increased between diagnosis and surgery in patients with complete regression and not in those with incomplete regression (29 samples from 13 patients; interaction coefficient − 1.70, *p* = 0.0002, q = 0.009). It was the only genus to remain significant after correction, and the result held when any single patient was omitted (largest *p* = 0.002). Ten further genera reached an uncorrected p below 0.05 (q ≥ 0.056) (Fig. 3a).

*Roseburia* moved in the same direction (interaction coefficient − 0.79, *p* = 0.085, q = 0.33). Before surgery it was more abundant in patients with complete regression (mean relative abundance 8.3% versus 1.0%; exact Mann–Whitney *p* = 0.057, four versus four patients with an evaluable pre-surgical sample, q = 1.00 across 84 genera). Two of the four complete responders carried this difference (individual values 1.1%, 2.2%, 10.2% and 19.6%); omitting either raised the interaction p above 0.19 (Fig. 3b). *Bacteroides* showed no significant time-point × response interaction (coefficient − 0.27, *p* = 0.35, q = 0.71) and is therefore reported as a descriptive observation only.

Because two of the 17 neoadjuvant patients received chemotherapy without radiotherapy, all longitudinal analyses were repeated excluding these two patients (both graded as incomplete regression in this cohort). All four alpha-diversity indices remained significantly associated with time point (likelihood-ratio *p* ≤ 0.0036), and the pre-surgical *Roseburia* difference was unchanged (*p* = 0.057). The *Phascolarctobacterium* time-point × response interaction was likewise materially unchanged after exclusion (coefficient − 1.69, *p* = 0.0004, q = 0.021; 11 patients) and remained the only genus significant after correction. The exposure is therefore described as neoadjuvant therapy throughout.

### Stool and tumor microbiota in paired samples

**Fig. 4:**
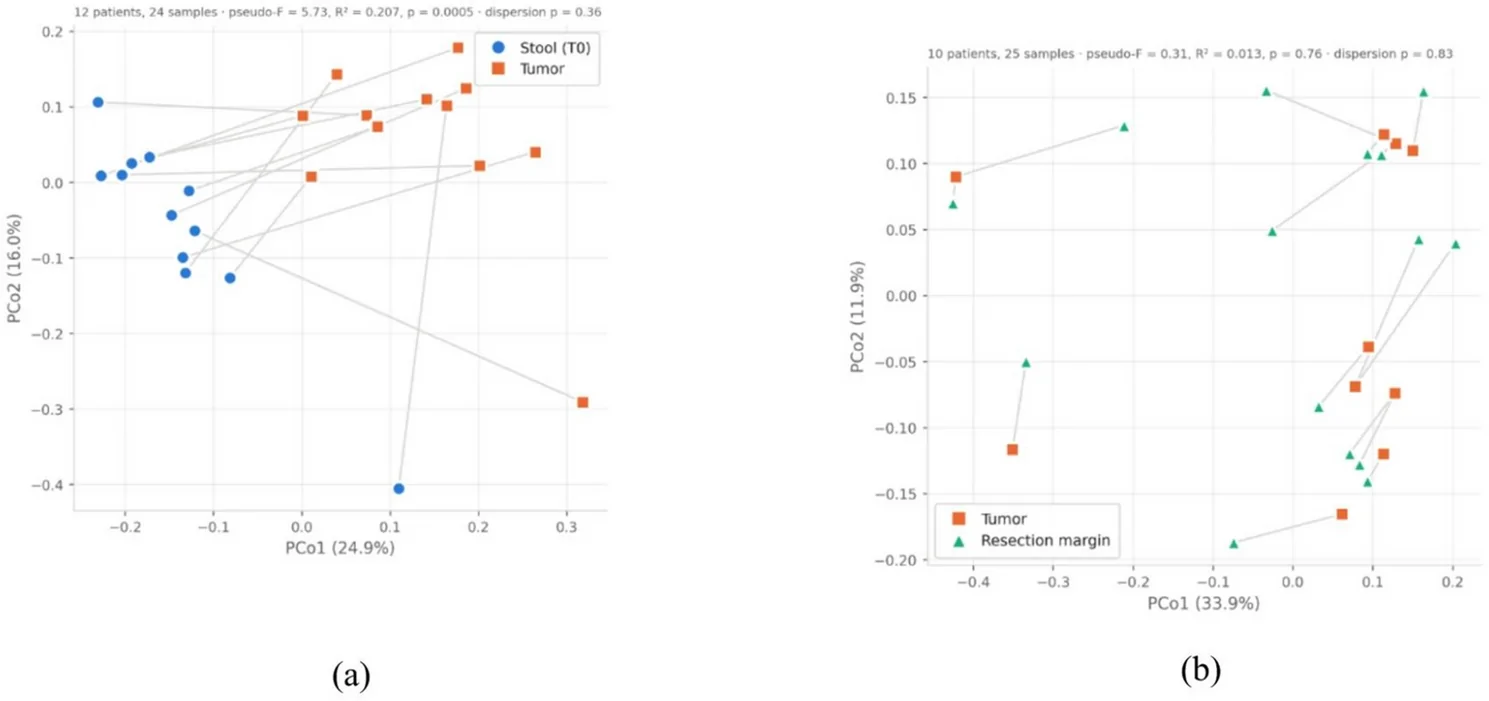
Paired compartment comparisons, genus level. PCoA of Bray–Curtis dissimilarities (log-transformed counts); grey lines connect samples from the same patient. **a **Native preoperative stool (T0) versus tumor tissue, 12 patients, 24 samples. **b** Tumor versus non-tumor resection-margin tissue, 10 patients, 25 samples, both processed with the same extraction kit and cycle number. PERMANOVA with within-patient permutations and betadisper; statistics above each panel

Preoperative stool samples (T0) were compared with paired tumor tissue in 12 patients. Community composition differed significantly between the two compartments (Bray–Curtis dissimilarity on log-transformed genus counts; PERMANOVA restricted to within-patient label exchanges, an exact paired test enumerating all 4,096 possible assignments: pseudo-F = 5.73, R² = 0.207, *p* = 0.0005). Multivariate dispersion did not differ between compartments (*p* = 0.36) (Fig. 4a).

At the genus level, an exact paired Wilcoxon signed-rank test identified 52 of 117 genera as differentially abundant between stool and tumor tissue following Benjamini–Hochberg correction.

Stool and non-tumor resection-margin tissue differed in the same way (10 patients, 25 samples; pseudo-F = 5.45, R² = 0.192, *p* = 0.0002, 9,999 permutations; dispersion *p* = 0.93). Tumor tissue and non-tumor resection-margin tissue did not differ (a different subset of 10 patients, 25 samples; pseudo-F = 0.31, R² = 0.013, *p* = 0.76; dispersion *p* = 0.83) (Fig. 4b).

### Tumor tissue microbiota by treatment group

**Fig. 5:**
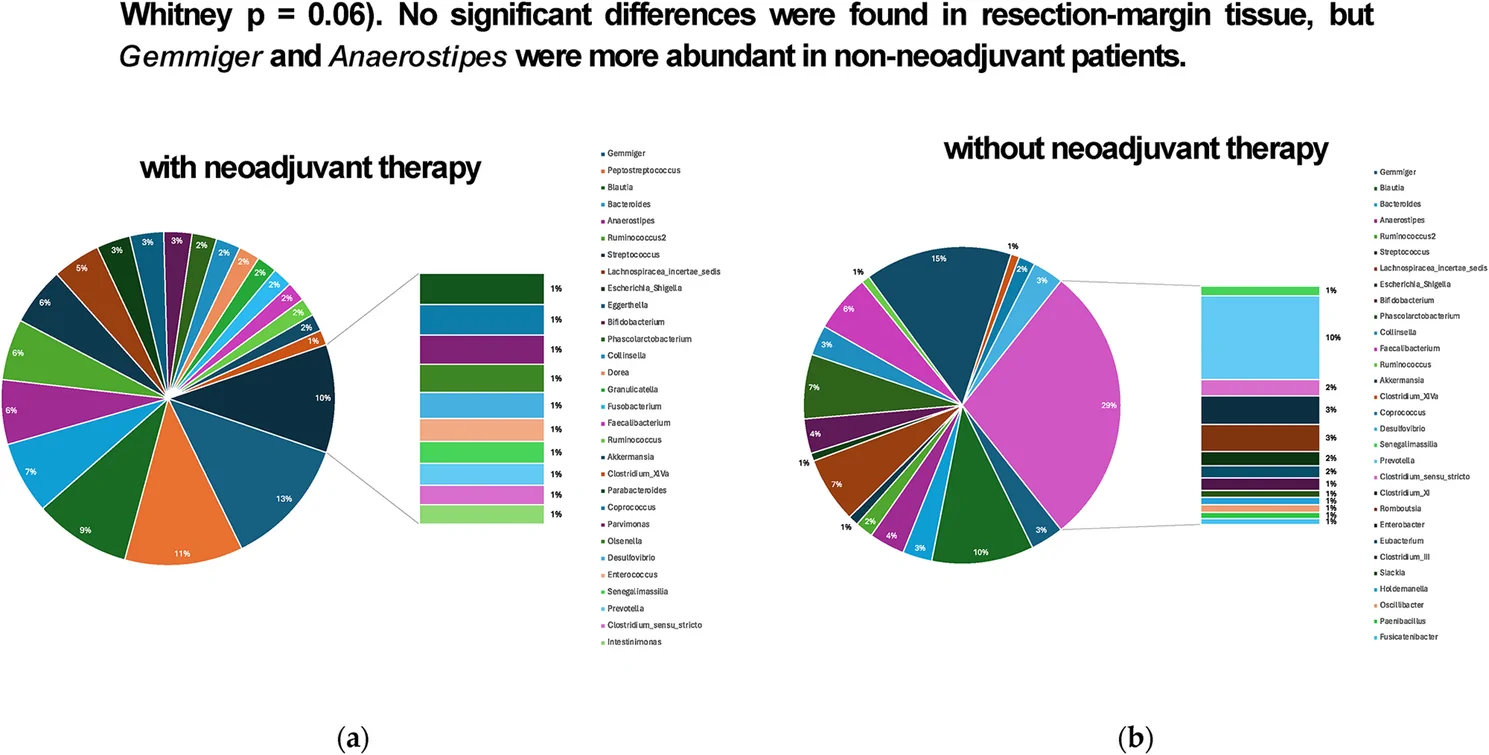
Comparison of tumor tissue microbiomes between non-neoadjuvant (**a**) and neoadjuvant-treated (**b**) patients. Relative genus-level abundance profiles show distinct microbial compositions between groups, with Gemmiger and Peptostreptococcus dominating in non-neoadjuvant and Akkermansia and Prevotella in neoadjuvant-treated patients

Tumor tissue microbiomes differed between neoadjuvant-treated and non-neoadjuvant patients (Fig. 5). Tumor tissue was available for microbiome analysis in 14 of the 31 patients (9 from the non-neoadjuvant group and 5 from the neoadjuvant group). This reduced number reflects the fact that diagnostic histopathology took precedence over research sampling. Tissue was retained for microbiome profiling only when sufficient material remained after routine diagnostic assessment (staging, tumor regression grading, and R-status). Specimens were taken from the macroscopically identified tumor or the tumor ulcer. Of the 5 neoadjuvant tumor samples, 2 originated from patients with complete pathological regression (TRG 4, representing treated tumor bed) and 3 from patients with incomplete regression (TRG 1–3). This sampling structure is independent of the longitudinal stool analysis, in which a higher number of patients could be included because stool collection did not depend on surgical resection. In the non-neoadjuvant group (*n* = 9), *Gemmiger*, *Peptostreptococcus*, and *Blautia* were prevalent (Fig. 5a), while *Akkermansia*, *Blautia*, and *Prevotella* dominated in the neoadjuvant-treated (*n* = 5) group (Fig. 5b). *Peptostreptococcus* was absent from all five neoadjuvant specimens and present in six of nine treatment-naïve specimens (exact Mann–Whitney *p* = 0.06). No significant differences were found in resection-margin tissue, but *Gemmiger* and *Anaerostipes* were more abundant in non-neoadjuvant patients.

## Discussion

Therapeutic response in rectal cancer remains highly variable despite standardized multimodal treatment strategies, and identifying biological determinants of response represents a major unmet clinical need. While the gut microbiome has emerged as a potential modulator of oncologic outcomes, most studies have examined colorectal cancer as a single entity or focused primarily on short-term perioperative changes. In this prospective study, we provide both a longitudinal analysis of fecal microbiome dynamics during neoadjuvant therapy and a compartment-resolved characterization of tissue-associated microbial communities in rectal cancer. By integrating serial stool sampling with tumor and resection margin tissue analyses, we relate microbial trajectories to neoadjuvant therapy and tumor regression while characterizing compartment-specific microbial profiles within the rectal tumor microenvironment.

A central finding of our study is the association between microbiome dynamics and complete tumor regression. Between diagnosis and surgery, the propionate-producing genus *Phascolarctobacterium* increased in patients achieving complete regression (Dworak TRG 4) but not in incomplete responders. This was the only genus to retain significance after correction for multiple testing and was stable when any single patient was omitted. A parallel but exploratory trend was observed for the butyrate-producing genus *Roseburia*, which shifted in the same direction and was more abundant before surgery in complete responders, whereas higher relative abundances of *Bacteroides* in incomplete responders were a descriptive observation that was not statistically significant. No genus differed between the two groups at baseline after correction for multiple testing; with only four complete and nine incomplete responders, however, the study has very limited power to exclude baseline differences, and a pre-existing signature therefore cannot be ruled out. Because 16 S rRNA sequencing characterizes taxonomy rather than function, and our responder subgroup was small, these associations remain exploratory. Nonetheless, they are consistent with experimental evidence linking short-chain fatty acid–producing bacteria to treatment response. Short-chain fatty acids such as propionate exert several potential anti-tumour effects, including epigenetic, pro-apoptotic and immunomodulatory actions; which of these, if any, underlies the association observed here cannot be determined from our data. *Phascolarctobacterium succinatutens* is known to produce propionate from succinate supplied by cross-feeding partners such as *Prevotella* [31], and propionate has recently been shown to enhance the efficacy of radiotherapy in colorectal cancer by promoting CD8 + T-cell infiltration [32]. In parallel, *Roseburia*-derived butyrate sensitizes colorectal cancer to radiotherapy through the butyrate/RALB axis and supports epithelial protection and anti-tumor CD8 + T-cell activity [13, 33, 34]. Conversely, *Bacteroides* has been associated with pro-inflammatory pathways and reduced radiosensitivity. Bacteroides vulgatus, for example, can promote resistance to chemotherapeutic stress through nucleotide biosynthesis [35]. Comparable associations between butyrate-producing bacteria and favorable chemoradiotherapy response have been reported previously [11, 12]. Together, these observations suggest a biologically plausible, but hypothetical, link between short-chain fatty acid production and response to neoadjuvant therapy. The clinical relevance of these findings extends beyond tumor regression alone. In rectal cancer, patients achieving a clinical complete response after neoadjuvant therapy may be eligible for a structured non-operative “watch-and-wait” strategy, enabling organ preservation. Given the substantial impact of rectal resection on bowel function, continence, and overall quality of life, identifying biological markers associated with complete response is clinical relevant. Although pathological complete response represents a favorable prognostic indicator, it does not fully eliminate the risk of distant metastatic spread. Continued surveillance and individualized long-term management therefore remain essential. Future studies should specifically investigate longitudinal microbiome signatures in patients managed with watch-and-wait strategies to determine whether fecal microbial patterns are associated with sustained complete response, local regrowth, or distant metastasis.

Longitudinal sampling showed that fecal microbial diversity declined during neoadjuvant chemoradiotherapy and did not recover before surgery (all four indices fell between T0 and T1; no index differed between T1 and T2, all q ≥ 0.72). Beyond this loss of richness, community composition itself shifted significantly over the treatment course in a patient-aware analysis (Bray–Curtis PERMANOVA with patient-constrained permutations, *p* = 0.0016), reflecting a change in community composition: *Oscillibacter* and *Lachnospiracea incertae sedis* progressively declined, whereas *Faecalibacterium* and *Clostridium XIVa persisted*,* the latter becoming the dominant genus before surgery*,* and Prevotella* emerged at the pre-surgical time point. This contraction is consistent with the reduction in microbial diversity commonly observed under cytotoxic and radiation therapy and implies that patients enter surgery with a reduced but compositionally restructured microbiome [36].

Postoperative samples showed the lowest microbial richness and evenness, consistent with previous reports describing a profound but transient dysbiosis following colorectal surgery [37–40]. This decline was also evident in stool with restored colonic passage (median observed richness 69.5) and, as expected, was most pronounced in ileostomy-derived samples (24.5). This reduction likely reflects the combined effects of bowel preparation, antibiotic prophylaxis, altered diet, and surgical stress on gut microbial ecology. This profound postoperative decline in microbial diversity aligns with experimental data demonstrating inflammation-driven disruption of microbial homeostasis and short-chain fatty acid depletion during early wound-healing phases [41]. The marked depletion of diversity underscores the vulnerability of the intestinal microbiome in the immediate postoperative period, which may contribute to impaired mucosal recovery or postoperative complications. Given the established role of short-chain fatty acids, particularly butyrate, in maintaining epithelial barrier integrity, regulating local immune responses, and supporting mucosal regeneration, their depletion may compromise intestinal healing processes [42–44]. This mechanism may partly explain why anastomotic complications can occur even in technically optimal settings, including tension-free anastomosis, adequate perfusion confirmed by intraoperative ICG fluorescence, and adherence to enhanced recovery protocols. Restoration of microbial balance through targeted nutritional or probiotic interventions could therefore represent an important adjunct to postoperative care.

Beyond treatment response, we observed pronounced spatial differences between luminal and tissue-associated microbiota. Stool samples were richer in *Bacteroidetes*, whereas tissue samples were dominated by *Firmicutes* and *Actinobacteria*. In tissue samples, *Actinobacteria* represented the second most abundant phylum (mean relative abundance approximately 12%), whereas *Proteobacteria* remained a minor component (approximately 3%). Moreover, preoperative stool harbored the most diverse microbial communities, whereas tissue compartments were intermediate and ileostomy-derived stool showed the lowest diversity. Beta diversity confirmed significant differences in microbial composition across compartments. Similar spatial segregation between luminal and tumor-associated microbiota has been described previously, suggesting distinct ecological niches within the colorectal environment [45–47]. Consistent with our observations, Kwon et al. reported that *Bacteroidetes* were significantly more abundant in stool than in tissue specimens, whereas *Firmicutes* and *Proteobacteria* were enriched in mucosal samples, indicating that stool may not fully capture mucosa-associated communities [48]. Within an individual tumor, by contrast, the microbiota has been reported to be spatially homogeneous, so that a single biopsy is representative of the lesion [49]. Compartment specific differences may influence the tumor microenvironment by modulating local immune cell infiltration and cytokine signaling, potentially affecting radiosensitivity and tissue repair [34, 44]. Interestingly, *Peptostreptococcus*, commonly associated with invasive biofilms, was absent from all neoadjuvant-treated tumor specimens while present in the majority of treatment-naïve samples [50, 51]. This is a descriptive observation in very small groups (nominal *p* = 0.06) obtained without extraction blanks and is not interpreted further.

This prospective study links longitudinal fecal microbiome dynamics during neoadjuvant therapy to tumor regression while resolving compartment-specific tissue communities in rectal cancer. A particular strength is the detailed characterization of the cohort, including dietary habits, alcohol and tobacco consumption, comorbidities, and medication use, which allowed us to characterize potential confounders of gut microbial composition. Several limitations should be acknowledged. First, as this was an exploratory study, the small number of patients with complete tumor regression (*n* = 4) limits the statistical robustness and generalizability of the response subgroup analyses. Second, treatment allocation was not randomized but followed clinical indications, resulting in confounding by indication. Neoadjuvant therapy was preferentially administered to patients with locally advanced mid- or low-rectal tumors, whereas patients with earlier-stage or more proximal tumors generally underwent primary surgery. Stool and tissue samples were processed with different DNA-extraction kits and amplification intensities (25 versus 35 PCR cycles). The marked compositional differences between stool and tissue are consistent with the well-established distinction between fecal and mucosa-associated communities, but technical variation may have contributed to their magnitude and to individual taxon-level estimates. No-template PCR controls were processed alongside the samples and yielded no reads, although dedicated extraction blanks were not included. Furthermore, the use of 16 S rDNA sequencing, while valuable for taxonomic profiling, does not capture functional microbial changes, limiting its insight into pathway-level alterations, metabolic capacity, and host–microbe interactions that may be directly relevant to treatment response.

Integrating functional approaches such as shotgun metagenomics, meta-transcriptomics, or metabolomics would therefore be essential to move beyond community composition and towards mechanistic understanding [52]. Because response was associated with short-chain fatty acid–producing taxa, a logical next step is whether these bacteria can be therapeutically enriched. Future studies should therefore explore microbiome-based interventions such as dietary modulation, probiotics, or fecal microbiota transplantation to enhance treatment response and recovery after nCRT. High-fiber strategies, for example, have been proposed to promote short-chain fatty acid–producing taxa, and diet-driven microbiome modulation is a growing focus in cancer therapy [53]. To our knowledge, no study has yet specifically investigated enriching these bacteria prior to neoadjuvant therapy in rectal cancer. Consistent with our findings, combined tumor-associated microbiome and immune profiling has recently been shown to predict response to neoadjuvant chemoradiotherapy in locally advanced rectal cancer [54]. Ultimately, prospectively validated microbiome signatures that identify patients likely to achieve a complete response could help select candidates for non-operative, organ-preserving watch-and-wait management and spare them the morbidity of radical surgery.

## Conclusions

This prospective longitudinal study demonstrates that rectal cancer treatment, particularly neoadjuvant chemoradiotherapy, induces pronounced and time-dependent alterations in the gut microbiome. While overall microbial diversity declined during therapy and did not recover before surgery, complete tumor regression was associated with an increase in the propionate-producing genus *Phascolarctobacterium*, the only genus to remain significant after correction for multiple testing, and, as an exploratory trend, with a higher pre-surgical abundance of the butyrate-producing genus *Roseburia*. This preliminary finding requires validation in larger studies. Higher *Bacteroides* in incomplete responders was a descriptive observation that was not statistically significant. Given the growing clinical relevance of non-operative management strategies, microbial correlates of tumor regression warrant investigation in larger cohorts; the present data are exploratory and do not establish a predictive biomarker.

## Supplementary information

Below is the link to the electronic supplementary material.

Supplementary Material 1: Figure S1 Phylum-level composition of stool and tissue sapmples

Supplementary Material 2: Figure S2 Genus-level microbiome composition of tumor, proximal, and distal tissue samples

Supplementary Material 3: Additional file 2: Table S1. Sample accounting. Table S1a: sample-level listing of all 110 samples. Table S1b: samples by group and time-point/compartment, including exclusions below 1,000 reads. Table S1c: samples and patients included in each analysis.

Supplementary Material 4: Figure S5 Core genus prevalence across detection thresholds in stoolsamples

Supplementary Material 5: Figure S6 Phylum-level composition of each individual sample

Supplementary Material 6: Figure S3 Sensitivity analysis of beta diversity

Supplementary Material 7: Figure S7 Rarefaction curves for each sample

## Data Availability

The datasets generated and analysed during the current study are available in the NCBI Sequence Read Archive (SRA) repository under BioProject accession PRJNA1495344 (https://www.ncbi.nlm.nih.gov/bioproject/PRJNA1495344).

## Abbreviations

APR: Abdominoperineal resection
BMI: Body Mass Index
CRC: Colorectal cancer
nCRT: Neoadjuvant chemoradiotherapy
5-FU: 5-Fluorouracil
OTUs: Operational taxonomic units
PCoA: Principal Coordinates Analysis
pCR: Pathological complete response
PERMANOVA: Permutational multivariate analysis of variance
TRG: Tumor regression grade
