# Supplementary material for: Longitudinal gut microbiome dynamics in rectal cancer: associations with neoadjuvant therapy and tumor regression
Source: BMC Microbiol. 2026 Nov 4;26:973. doi: 10.1186/s12866-026-05859-y (PMC13741762; doi:10.1186/s12866-026-05859-y)
Supplement: Supplementary file 1 [file 12866_2026_5859_MOESM1_ESM.pdf]

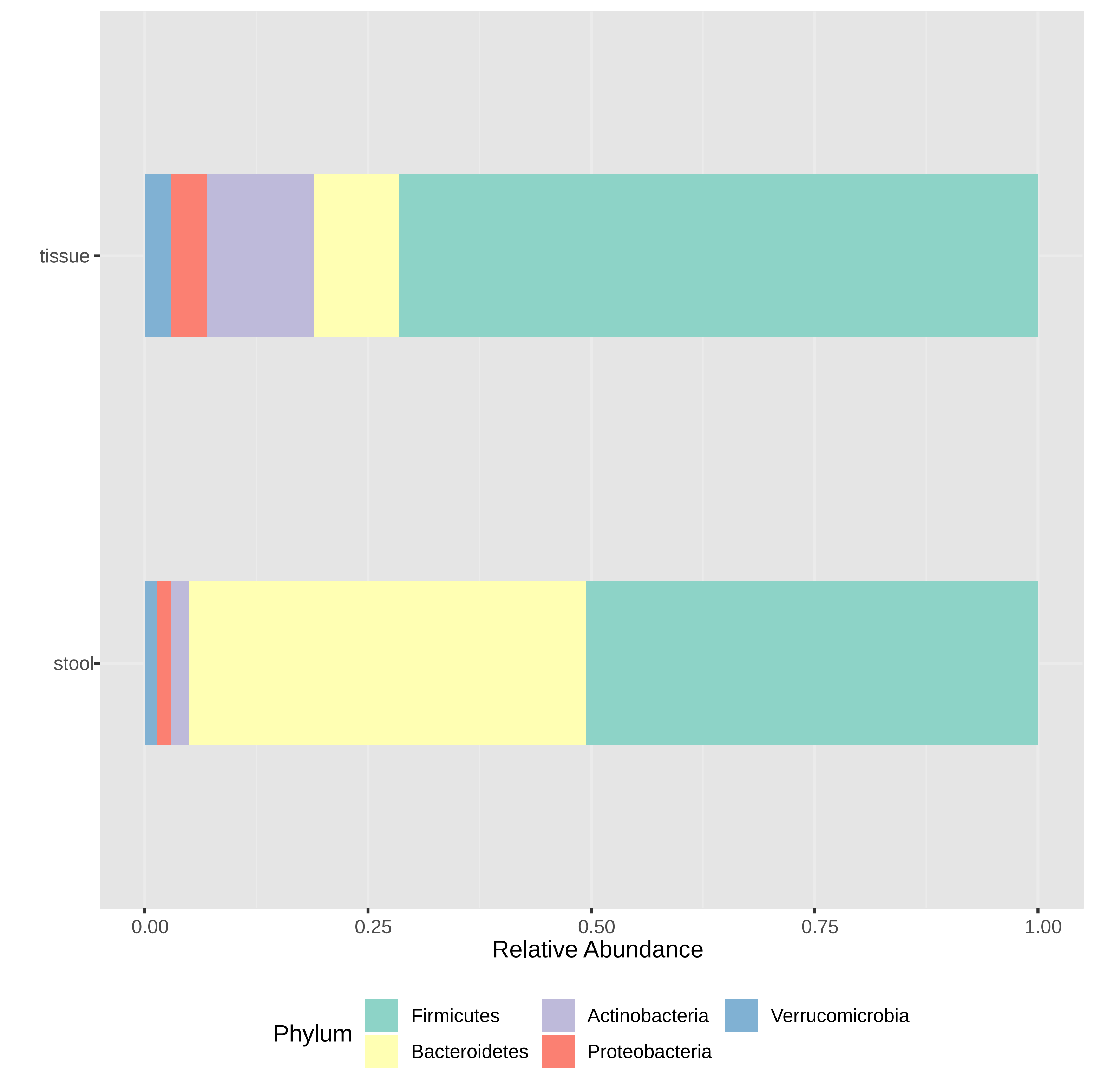
tissue
stool
0.00
0.25
0.50
0.75
1.00
Relative Abundance
Phylum
Firmicutes
Bacteroidetes
Actinobacteria
Proteobacteria
Verrucomicrobia
