# Supplementary material for: Longitudinal gut microbiome dynamics in rectal cancer: associations with neoadjuvant therapy and tumor regression
Source: BMC Microbiol. 2026 Nov 4;26:973. doi: 10.1186/s12866-026-05859-y (PMC13741762; doi:10.1186/s12866-026-05859-y)
Supplement: Supplementary file 2 [file 12866_2026_5859_MOESM2_ESM.pdf]

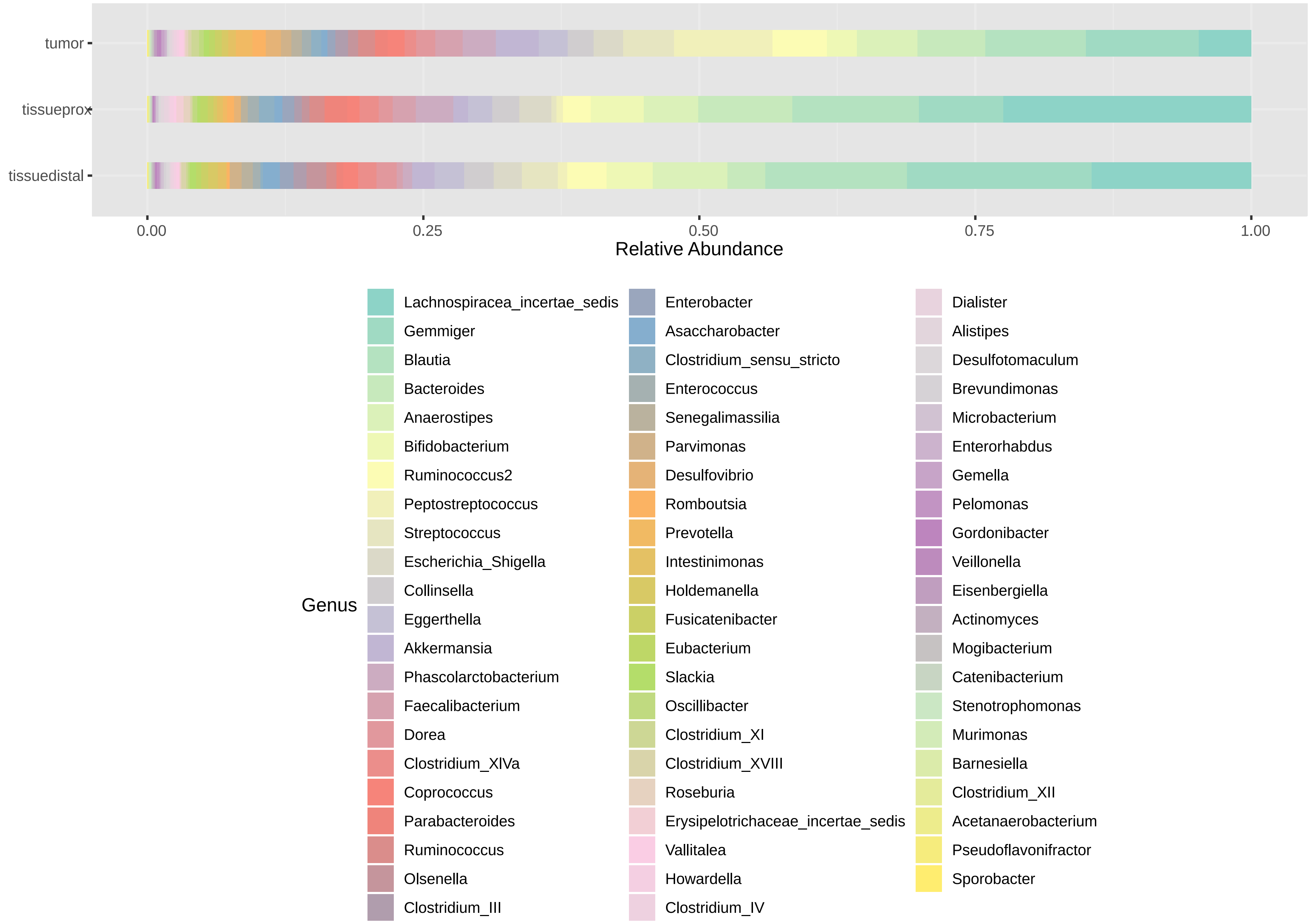
tumor
tissueprox
tissuedistal
0.00
0.25
0.50
0.75
1.00
Relative Abundance
Genus
Lachnospiracea_incertae_sedis
Gemmiger
Blautia
Bacteroides
Anaerostipes
Bifidobacterium
Ruminococcus2
Peptostreptococcus
Streptococcus
Escherichia_Shigella
Collinsella
Eggerthella
Akkermansia
Phascolarctobacterium
Faecalibacterium
Dorea
Clostridium_XlVa
Coprococcus
Parabacteroides
Ruminococcus
Olsenella
Clostridium_III
Enterobacter
Asaccharobacter
Clostridium_sensu_stricto
Enterococcus
Senegalimassilia
Parvimonas
Desulfovibrio
Romboutsia
Prevotella
Intestinimonas
Holdemanella
Fusicatenibacter
Eubacterium
Slackia
Oscillibacter
Clostridium_XI
Clostridium_XVIII
Roseburia
Erysipelotrichaceae_incertae_sedis
Vallitalea
Howardella
Clostridium_IV
Dialister
Alistipes
Desulfotomaculum
Brevundimonas
Microbacterium
Enterorhabdus
Gemella
Pelomonas
Gordonibacter
Veillonella
Eisenbergiella
Actinomyces
Mogibacterium
Catenibacterium
Stenotrophomonas
Murimonas
Barnesiella
Clostridium_XII
Acetanaerobacterium
Pseudoflavonifractor
Sporobacter
