# Supplementary material for: Longitudinal gut microbiome dynamics in rectal cancer: associations with neoadjuvant therapy and tumor regression
Source: BMC Microbiol. 2026 Nov 4;26:973. doi: 10.1186/s12866-026-05859-y (PMC13741762; doi:10.1186/s12866-026-05859-y)
Supplement: Supplementary file 3 [file 12866_2026_5859_MOESM3_ESM.pdf]

Table S1. Sample accounting.

Table S1b. Samples by group and time-point/compartment (QC: minimum 1,000 reads).

Group	Timepoint/Compartment	Samples collected	Patients	Median reads	Excluded <1000 reads	Samples analysed	Patients analysed
Neoadjuvant (n=17)	T0	15	15	12616	1	14	14
Neoadjuvant (n=17)	T1	12	12	2896	0	12	12
Neoadjuvant (n=17)	T2	10	10	5468	1	9	9
Neoadjuvant (n=17)	T3	2	2	7411	0	2	2
Neoadjuvant (n=17)	T3IS	4	4	5255	0	4	4
Neoadjuvant (n=17)	tumor	5	5	4623	0	5	5
Neoadjuvant (n=17)	prox	7	7	1043	3	4	4
Neoadjuvant (n=17)	distal	3	3	30	2	1	1
Primary surgery (n=14)	preop_pR	13	13	15154	0	13	13
Primary surgery (n=14)	T3	5	5	4445	1	4	4
Primary surgery (n=14)	T3IS	8	8	4715	1	7	7
Primary surgery (n=14)	tumor	9	9	13206	0	9	9
Primary surgery (n=14)	prox	8	8	11707	2	6	6
Primary surgery (n=14)	distal	9	9	7797	2	7	7

Table S1c. Samples and patients included in each analysis.

Analysis	Included
Alpha diversity T0 vs T1 (paired Wilcoxon)	9 patients (paired), rarefied to 1,300 reads
Alpha diversity T1 vs T2 (paired Wilcoxon)	8 patients (paired)
Alpha diversity T0 vs T2 (paired Wilcoxon)	7 patients (paired)
PERMANOVA longitudinal + dispersion (strata=patient)	35 samples / 17 patients
Responder analysis T2, genus level (exact MW + BH-FDR)	CR 4 vs IR 4 patients (8 samples); 1 IR sample excluded (19 reads)
Sensitivity: nCRT only (excl. 2 CTx-only patients)	15 patients
Postoperative comparison T3 vs T3IS	T3 6 vs T3IS 11 samples
Tumour tissue nT vs pR (Peptostreptococcus, exact MW)	5 vs 9 samples (all depths; result robust to QC)

Notes: Rarefaction depth 1,300 reads = the minimum sequencing depth (lowest included stool sample). Paired analyses include only patients with both samples available. The two patients who received chemotherapy without radiotherapy were both incomplete responders; the complete-response group therefore consists exclusively of nCRT-treated patients.

Table S1a. Sample-level listing of all 110 samples.

Sample	patient	grp	therapy	tp	resp	reads	QC1000
Sample481	P06	nT	nCRT	T0	IR	18131	included
Sample494	P11	nT	nCRT	T0	CR	2336	included
Sample477	P14	nT	CTx only	T0	IR	62	excluded
Sample486	P15	nT	nCRT	T0	CR	20451	included
Sample487	P16	nT	nCRT	T0	IR	15001	included
Sample479	P17	nT	nCRT	T0	CR	21644	included
Sample485	P18	nT	nCRT	T0	IR	23750	included
Sample503	P19	nT	nCRT	T0	CR	26366	included
Sample489	P21	nT	nCRT	T0	IR	18097	included
Sample497	P22	nT	nCRT	T0	IR	2807	included
Sample496	P23	nT	nCRT	T0	NA	1366	included
Sample493	P24	nT	nCRT	T0	NA	2118	included
Sample490	P29	nT	nCRT	T0	IR	12616	included
Sample499	P30	nT	nCRT	T0	NA	9403	included
Sample502	P31	nT	nCRT	T0	NA	1541	included
Sample437	P06	nT	nCRT	T1	IR	4957	included
Sample438	P10	nT	nCRT	T1	IR	2783	included
Sample443	P11	nT	nCRT	T1	CR	2970	included
Sample435	P14	nT	CTx only	T1	IR	1300	included
Sample446	P15	nT	nCRT	T1	CR	23308	included
Sample436	P17	nT	nCRT	T1	CR	2939	included
Sample439	P18	nT	nCRT	T1	IR	2625	included
Sample440	P19	nT	nCRT	T1	CR	2220	included
Sample442	P20	nT	CTx only	T1	IR	2854	included
Sample444	P21	nT	nCRT	T1	IR	3831	included
Sample445	P22	nT	nCRT	T1	IR	2376	included
Sample447	P23	nT	nCRT	T1	NA	3536	included
Sample450	P06	nT	nCRT	T2	IR	3249	included
Sample453	P10	nT	nCRT	T2	IR	2140	included
Sample448	P11	nT	nCRT	T2	CR	2013	included
Sample452	P14	nT	CTx only	T2	IR	11214	included

Sample	patient	grp	therapy	tp	resp	reads	QC1000
Sample449	P15	nT	nCRT	T2	CR	12622	included
Sample455	P16	nT	nCRT	T2	IR	19	excluded
Sample441	P17	nT	nCRT	T2	CR	3700	included
Sample454	P18	nT	nCRT	T2	IR	7237	included
Sample456	P19	nT	nCRT	T2	CR	9087	included
Sample457	P24	nT	nCRT	T2	NA	12916	included
Sample463	P15	nT	nCRT	T3	CR	11635	included
Sample451	P17	nT	nCRT	T3	CR	3187	included
Sample466	P06	nT	nCRT	T3IS	IR	3087	included
Sample469	P11	nT	nCRT	T3IS	CR	1194	included
Sample473	P18	nT	nCRT	T3IS	IR	7423	included
Sample474	P29	nT	nCRT	T3IS	IR	12419	included
Sample399	P06	nT	nCRT	distal	IR	4	excluded
Sample403	P10	nT	nCRT	distal	IR	1240	included
Sample404	P11	nT	nCRT	distal	CR	30	excluded
Sample414	P06	nT	nCRT	prox	IR	3	excluded
Sample419	P10	nT	nCRT	prox	IR	12017	included
Sample420	P11	nT	nCRT	prox	CR	1606	included
Sample412	P14	nT	CTx only	prox	IR	8	excluded
Sample413	P15	nT	nCRT	prox	CR	6327	included
Sample416	P16	nT	nCRT	prox	IR	1	excluded
Sample418	P17	nT	nCRT	prox	CR	1043	included
Sample428	P06	nT	nCRT	tumor	IR	1708	included
Sample432	P10	nT	nCRT	tumor	IR	1427	included
Sample433	P11	nT	nCRT	tumor	CR	5330	included
Sample427	P15	nT	nCRT	tumor	CR	10457	included
Sample431	P18	nT	nCRT	tumor	IR	4623	included
Sample459	P01	pR	none (primary surgery)	T3	NA	32797	included
Sample462	P03	pR	none (primary surgery)	T3	NA	25341	included
Sample471	P08	pR	none (primary surgery)	T3	NA	1572	included
Sample472	P09	pR	none (primary surgery)	T3	NA	4445	included
Sample468	P28	pR	none (primary surgery)	T3	NA	919	excluded
Sample458	P02	pR	none (primary surgery)	T3IS	NA	23827	included

Sample	patient	grp	therapy	tp	resp	reads	QC1000
Sample460	P04	pR	none (primary surgery)	T3IS	NA	63	excluded
Sample475	P05	pR	none (primary surgery)	T3IS	NA	6808	included
Sample470	P07	pR	none (primary surgery)	T3IS	NA	1008	included
Sample461	P13	pR	none (primary surgery)	T3IS	NA	18855	included
Sample464	P25	pR	none (primary surgery)	T3IS	NA	12597	included
Sample465	P26	pR	none (primary surgery)	T3IS	NA	2545	included
Sample467	P27	pR	none (primary surgery)	T3IS	NA	2623	included
Sample394	P01	pR	none (primary surgery)	distal	NA	12182	included
Sample395	P02	pR	none (primary surgery)	distal	NA	10509	included
Sample396	P03	pR	none (primary surgery)	distal	NA	21050	included
Sample397	P04	pR	none (primary surgery)	distal	NA	7797	included
Sample398	P05	pR	none (primary surgery)	distal	NA	25837	included
Sample400	P07	pR	none (primary surgery)	distal	NA	6348	included
Sample401	P08	pR	none (primary surgery)	distal	NA	4	excluded
Sample402	P09	pR	none (primary surgery)	distal	NA	1	excluded
Sample405	P12	pR	none (primary surgery)	distal	NA	1406	included
Sample478	P01	pR	none (primary surgery)	preop_pR	NA	31205	included
Sample476	P02	pR	none (primary surgery)	preop_pR	NA	45651	included
Sample483	P03	pR	none (primary surgery)	preop_pR	NA	17462	included
Sample480	P04	pR	none (primary surgery)	preop_pR	NA	20678	included
Sample484	P05	pR	none (primary surgery)	preop_pR	NA	26314	included
Sample498	P07	pR	none (primary surgery)	preop_pR	NA	3097	included
Sample500	P08	pR	none (primary surgery)	preop_pR	NA	7711	included
Sample501	P09	pR	none (primary surgery)	preop_pR	NA	4621	included
Sample482	P13	pR	none (primary surgery)	preop_pR	NA	15154	included
Sample488	P25	pR	none (primary surgery)	preop_pR	NA	17877	included
Sample491	P26	pR	none (primary surgery)	preop_pR	NA	2119	included
Sample492	P27	pR	none (primary surgery)	preop_pR	NA	2439	included
Sample495	P28	pR	none (primary surgery)	preop_pR	NA	2316	included
Sample406	P01	pR	none (primary surgery)	prox	NA	12753	included
Sample407	P02	pR	none (primary surgery)	prox	NA	20412	included
Sample408	P03	pR	none (primary surgery)	prox	NA	15288	included
Sample409	P04	pR	none (primary surgery)	prox	NA	6	excluded

Sample	patient	grp	therapy	tp	resp	reads	QC1000
Sample411	P05	pR	none (primary surgery)	prox	NA	24989	included
Sample415	P07	pR	none (primary surgery)	prox	NA	3304	included
Sample417	P09	pR	none (primary surgery)	prox	NA	2	excluded
Sample410	P13	pR	none (primary surgery)	prox	NA	10661	included
Sample421	P01	pR	none (primary surgery)	tumor	NA	13206	included
Sample422	P02	pR	none (primary surgery)	tumor	NA	6639	included
Sample423	P03	pR	none (primary surgery)	tumor	NA	22980	included
Sample424	P04	pR	none (primary surgery)	tumor	NA	21381	included
Sample426	P05	pR	none (primary surgery)	tumor	NA	20747	included
Sample429	P08	pR	none (primary surgery)	tumor	NA	5373	included
Sample430	P09	pR	none (primary surgery)	tumor	NA	5875	included
Sample434	P12	pR	none (primary surgery)	tumor	NA	1543	included
Sample425	P13	pR	none (primary surgery)	tumor	NA	18625	included
