# Supplementary material for: Longitudinal gut microbiome dynamics in rectal cancer: associations with neoadjuvant therapy and tumor regression
Source: BMC Microbiol. 2026 Nov 4;26:973. doi: 10.1186/s12866-026-05859-y (PMC13741762; doi:10.1186/s12866-026-05859-y)

(a) T0 — diagnosis

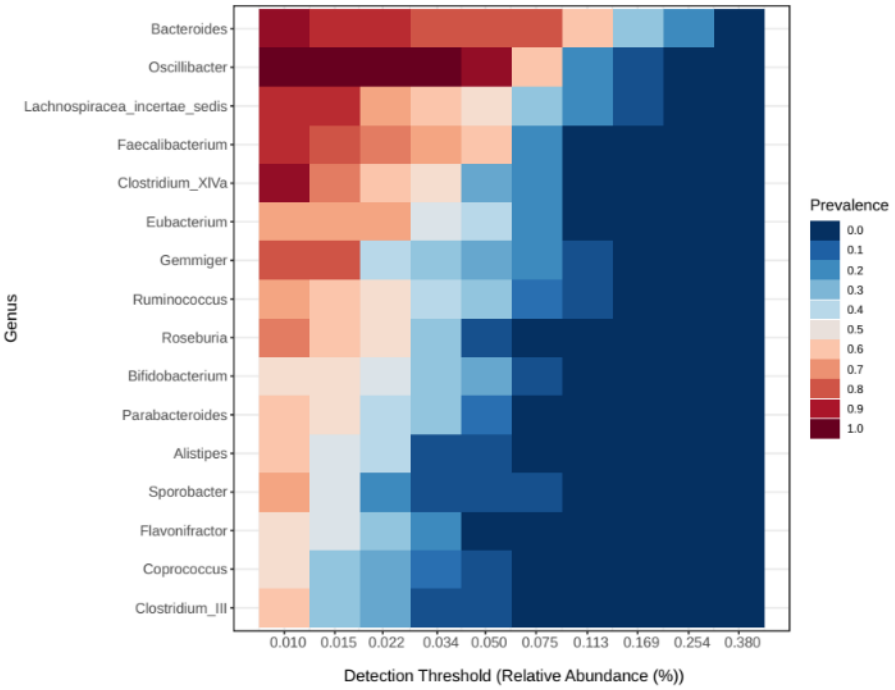

(b) T1 — after nCRT

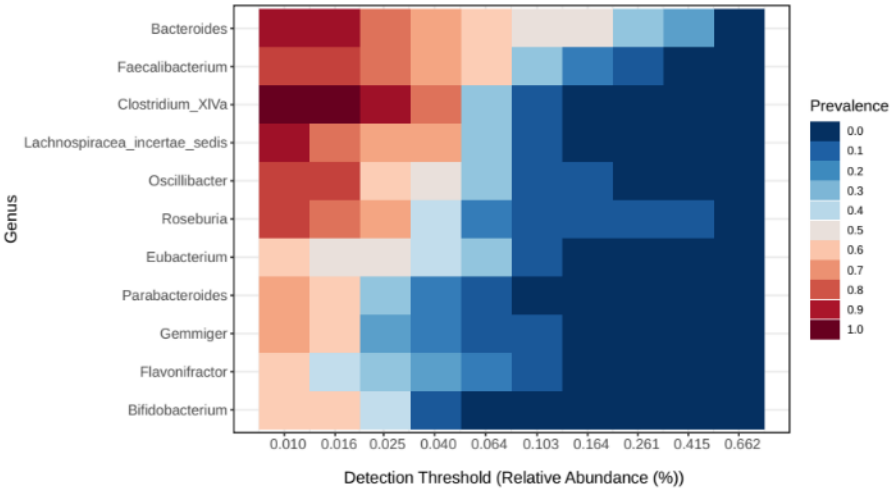

(c) T2 — before surgery

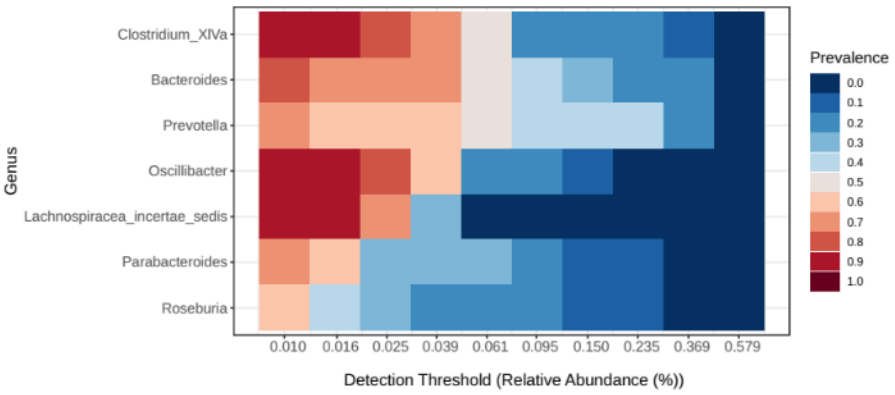

Supplement: Supplementary file 4 [file 12866_2026_5859_MOESM4_ESM.pdf]
