# Supplementary material for: Longitudinal gut microbiome dynamics in rectal cancer: associations with neoadjuvant therapy and tumor regression
Source: BMC Microbiol. 2026 Nov 4;26:973. doi: 10.1186/s12866-026-05859-y (PMC13741762; doi:10.1186/s12866-026-05859-y)
Supplement: Supplementary file 5 [file 12866_2026_5859_MOESM5_ESM.pdf]

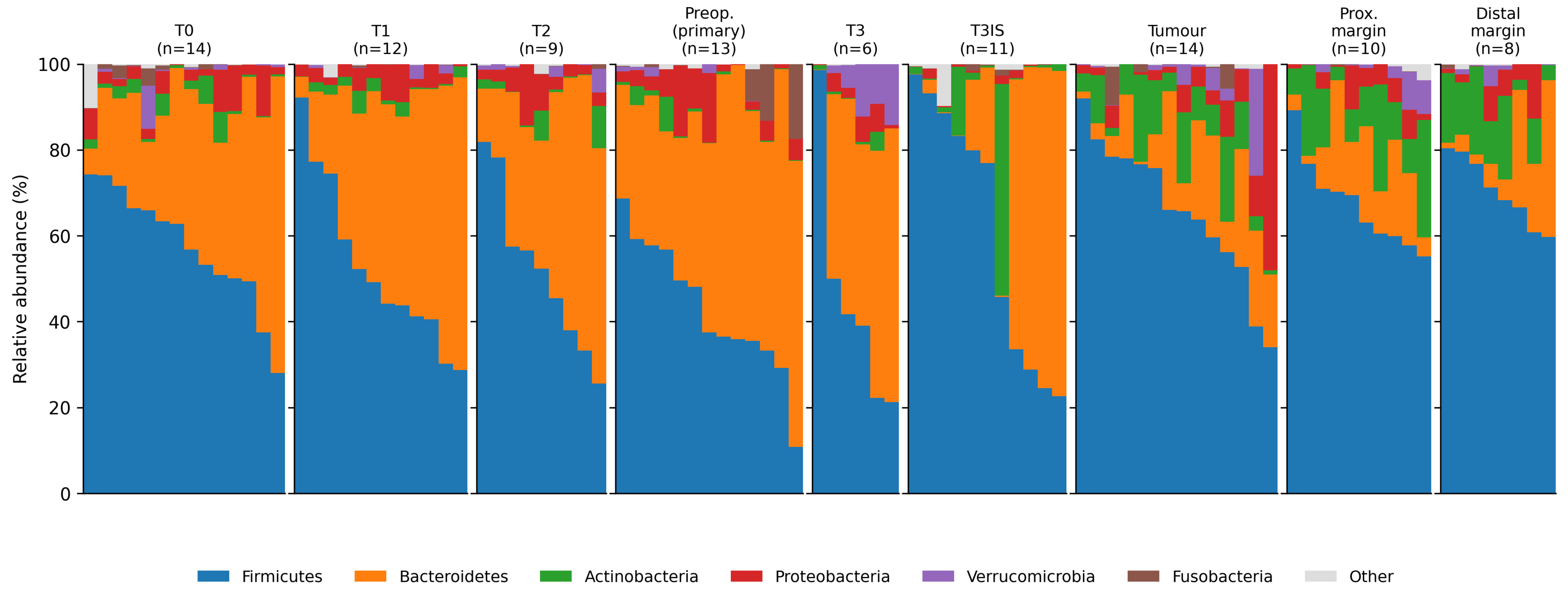
T0 (n=14)
T1 (n=12)
T2 (n=9)
Preop. (primary) (n=13)
T3 (n=6)
T3IS (n=11)
Tumour (n=14)
Prox. margin (n=10)
Distal margin (n=8)
Relative abundance (%)
0
20
40
60
80
100
Firmicutes
Bacteroidetes
Actinobacteria
Proteobacteria
Verrucomicrobia
Fusobacteria
Other
