# Supplementary material for: Longitudinal gut microbiome dynamics in rectal cancer: associations with neoadjuvant therapy and tumor regression
Source: BMC Microbiol. 2026 Nov 4;26:973. doi: 10.1186/s12866-026-05859-y (PMC13741762; doi:10.1186/s12866-026-05859-y)
Supplement: Supplementary file 6 [file 12866_2026_5859_MOESM6_ESM.pdf]

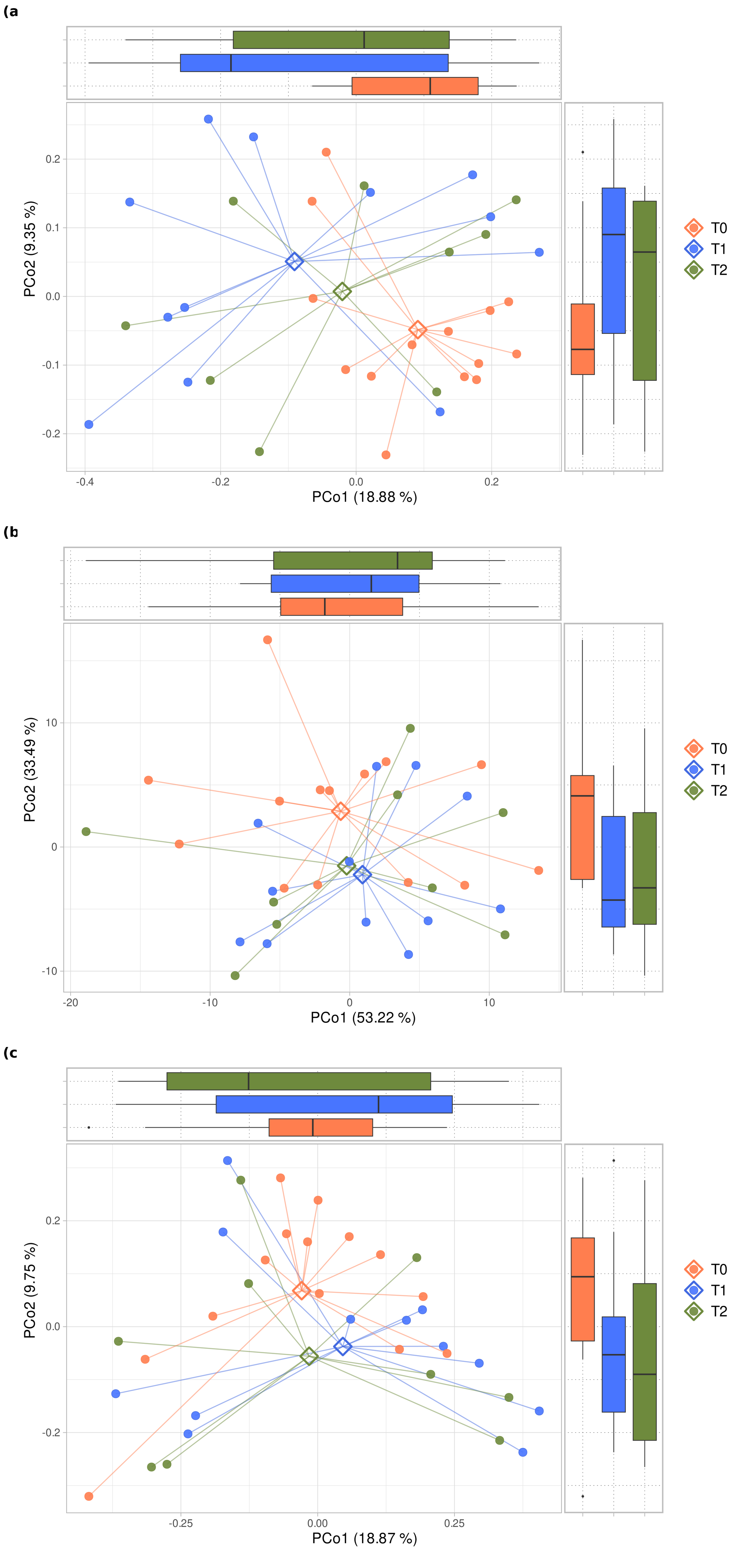

(a
PCo2 (9.35 %)
0.2
0.1
0.0
-0.1
-0.2
-0.4
-0.2
0.0
0.2
PCo1 (18.88 %)
T0
T1
T2
(b
PCo2 (33.49 %)
10
0
-10
-20
-10
0
10
PCo1 (53.22 %)
T0
T1
T2
(c
PCo2 (9.75 %)
0.2
0.0
-0.2
-0.25
0.00
0.25
PCo1 (18.87 %)
T0
T1
T2
