# Supplementary material for: Longitudinal gut microbiome dynamics in rectal cancer: associations with neoadjuvant therapy and tumor regression
Source: BMC Microbiol. 2026 Nov 4;26:973. doi: 10.1186/s12866-026-05859-y (PMC13741762; doi:10.1186/s12866-026-05859-y)
Supplement: Supplementary file 7 [file 12866_2026_5859_MOESM7_ESM.pdf]

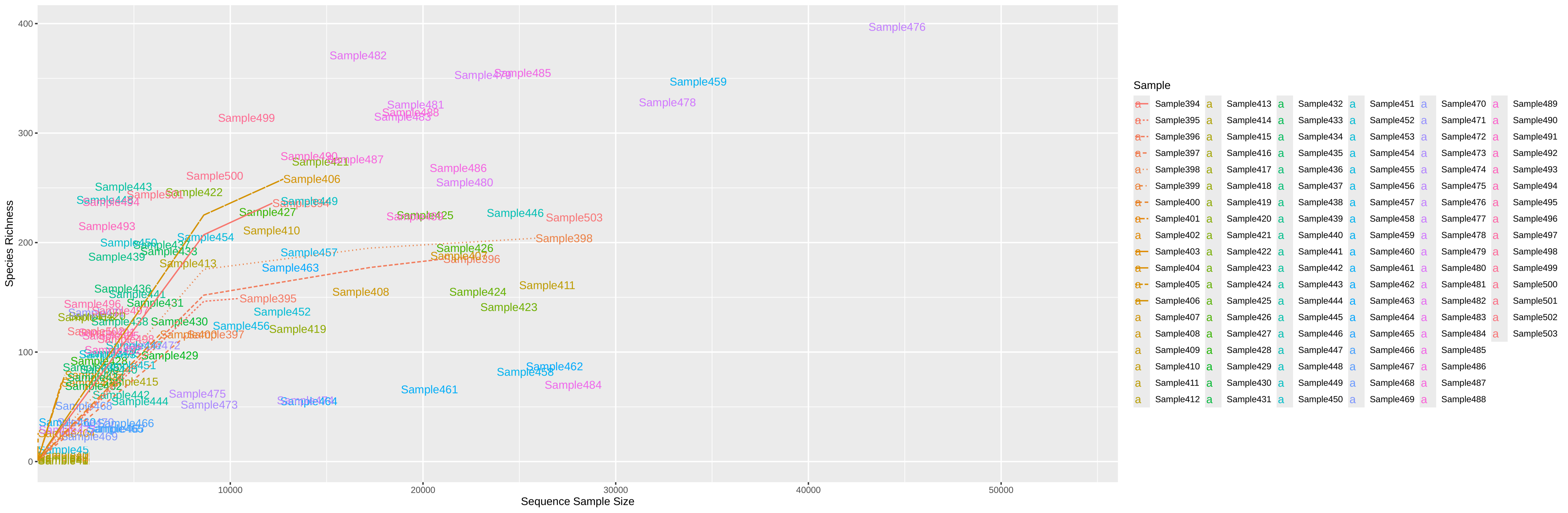

Species Richness
400
300
200
100
0
10000
20000
30000
40000
50000
Sequence Sample Size
Sample
Sample394
Sample395
Sample396
Sample397
Sample398
Sample399
Sample400
Sample401
Sample402
Sample403
Sample404
Sample405
Sample406
Sample407
Sample408
Sample409
Sample410
Sample411
Sample412
Sample413
Sample414
Sample415
Sample416
Sample417
Sample418
Sample419
Sample420
Sample421
Sample422
Sample423
Sample424
Sample425
Sample426
Sample427
Sample428
Sample429
Sample430
Sample431
Sample432
Sample433
Sample434
Sample435
Sample436
Sample437
Sample438
Sample439
Sample440
Sample441
Sample442
Sample443
Sample444
Sample445
Sample446
Sample447
Sample448
Sample449
Sample450
Sample451
Sample452
Sample453
Sample454
Sample455
Sample456
Sample457
Sample458
Sample459
Sample460
Sample461
Sample462
Sample463
Sample464
Sample465
Sample466
Sample467
Sample468
Sample469
Sample470
Sample471
Sample472
Sample473
Sample474
Sample475
Sample476
Sample477
Sample478
Sample479
Sample480
Sample481
Sample482
Sample483
Sample484
Sample485
Sample486
Sample487
Sample488
Sample489
Sample490
Sample491
Sample492
Sample493
Sample494
Sample495
Sample496
Sample497
Sample498
Sample499
Sample500
Sample501
Sample502
Sample503
